# Impact of 13-valent pneumococcal conjugate vaccine on laboratory-confirmed pneumococcal meningitis and purulent meningitis among children ˂5 years in Cameroon, 2011–2018

**DOI:** 10.1371/journal.pone.0250010

**Published:** 2021-04-15

**Authors:** John Njuma Libwea, Mark A. Fletcher, Paul Koki Ndombo, Angeline Boula, Nadesh Taku Ashukem, Madeleine Ngo Baleba, Rachel Sandrine Kingue Bebey, Eric Gaston Nkolo Mviena, Jean Tageube, Marie Kobela Mbollo, Sinata Koulla-Shiro, Shabir Madhi, Berthe-Marie Njanpop-Lafourcade, Ali Mohammad, Elizabeth Begier, Joanna Southern, Rohini Beavon, Bradford Gessner

**Affiliations:** 1 National Institute for Health and Welfare (THL), Helsinki, Finland; 2 Health Sciences Unit, Faculty of Social Sciences, Tampere University, Tampere, Finland; 3 Expanded Programme on Immunization, Cameroon; 4 Emerging Markets Medical Affairs, Vaccines, Pfizer, Inc, Paris, France; 5 Mother & Child Hospital (MCH), Chantal Biya Foundation, Yaoundé, Cameroon; 6 Ministry of Public Health, Yaoundé, Cameroon; 7 Faculty of Medicine and Biomedical Sciences, University of Yaoundé 1, Yaoundé, Cameroon; 8 South African Medical Research Council Vaccines and Infectious Diseases Analytical Research Unit, Faculty of HealthSciences, University of the Witwatersrand, Johannesburg, South Africa; 9 Agence de Médicine Préventive (AMP), Paris, France; 10 Pfizer Vaccines Medical Development & Medical/Scientific Affairs, New York, New York, United States of America; Meyer Children’s University Hospital - University of Florence, ITALY

## Abstract

**Background:**

The 13-valent pneumococcal conjugate vaccine (PCV13) entered Cameroon’s childhood national immunization programme (NIP) in July 2011 under a 3-dose schedule (6, 10, 14 weeks of age) without any catch-up. We described the impact of PCV13 onserotype distribution among pneumococcal meningitis cases over time.

**Methods:**

We used laboratory-based sentinel surveillance data to identify meningitis cases among 2- to 59-month-old children with clinically-suspected bacterial meningitis (CSBM) admitted to hospitals in Yaoundé (August 2011-December 2018). Purulent meningitis cases had a cerebrospinal fluid (CSF) white blood cell (WBC) count ≥20 per mm^3^. Pneumococcal meningitis cases had *S*. *pneumoniae* identified from CSF, with serotyping by polymerase chain reaction. Years 2011-2014 were described as early PCV13 era (EPE) and years 2015-2018 as late PCV13 era (LPE) impact periods.

**Results:**

Among children hospitalized with CSBM who had a lumbar puncture obtained, there was no significant change from the EPE versus the LPE in the percentage identified with purulent meningitis: 7.5% (112/1486) versus 9.4% (154/1645), p = 0.0846. The percentage of pneumococcal meningitis cases due to PCV13 vaccine-serotype (VST) decreased from 62.0% (31/50) during the EPE to 35.8% (19/53) in the LPE, p = 0.0081. The most frequent pneumococcal meningitis VSTs during the EPE were 6A/6B (30%) and 5 (6%), and during the LPE were 14 (13.2%), 3 (7.6%), 4 (5.6%) and 18C (5.6%).

**Conclusion:**

Four to seven years after PCV13 introduction, the proportion of pneumococcal meningitis due to vaccine serotypes has declined, mainly due to reductions of serotypes 6A/6B, 1, 19A, and 23F; nevertheless, PCV13 VSTs remain common. Because the analyzed surveillance system was not consistent or population based, we could not estimate incidence or overall impact; this emphasizes the need for improved surveillance to document further the utility of PCV13 immunization in Cameroon.

## 1. Background

*Streptococcus pneumoniae* remains a major cause of illness and death in children and adults worldwide [1], with serious disease due to pneumonia, septicemia and meningitis [2]. Over 2 million children under the age of five years still die from pneumonia and related complications each year, two-thirds of these in developing countries [3]. The situation is precarious in Sub-Saharan Africa, especially among the poorest twentieth percentile of the population and among young children [4]. In Cameroon, the overall mortality rate among children age <5 years is 88/1000 live births, and 10–19 % of these deaths result from pneumonia-associated infections [3, 5, 6].

Pneumococcal conjugate vaccine (PCV) was first licensed as a 7-valent vaccine (PCV7). Subsequently 10- and 13-valent products (PCV10 and PCV13, respectively) became available. PCV7 was first introduced to infant national immunization programs (NIPs) in the United States in the year 2000, Europe in 2001 and Africa in 2009. Both PCV10 and PCV13, contain serotypes 1 and 5, which are prevalent in developing countries [7, 8], leading to expectations of relatively broad coverage in Sub-Saharan Africa.

The efficacy of PCVs in Sub-Saharan Africa was first assessed in South Africa (in a clinical trial with an investigational 9-valent PCV [9] and subsequently in the NIP with PCV7 transitioning later to PCV13), and in The Gambia (in a clinical trial of the investigational PCV9 and subsequently, in the national vaccination programme with PCV7 transitioning to PCV13) [6]. Data from these studies were supported by recent studies from Mozambique, Kenya, Burkina Faso and Senegal [10–13]. For example, the South African PCV9 trial reported an 83% reduction of IPD due to vaccine serotypes in HIV-negative participants [14], supporting the use of PCVs more broadly in Africa.

Before PCV13 introduction in Cameroon, Gervais et al. [15] demonstrated, among 170 Cameroonian children aged 2 months to 15 years hospitalized with meningitis, that PCV13-serotypes 1 (12 %), 6A (4 %), 14 (4 %), 5 (3 %), 7F (3 %) and 19A (3 %) and the non-PCV13 serotypes 32A/32F (8%) and 23B (4%) dominated. Subsequently, PCV13 was included in the NIP in Cameroon under a 3-dose schedule (6, 10 and 14 weeks of age; 3+0) without any catch-up doses for older children. PCV13 was administered jointly with a pentavalent vaccine (DTwP-Hib-HepB) including diphtheria, tetanus, whole cell pertussis, *Haemophilus influenzae* type b, and hepatitis B. Since 2012, as reported by the administration, uptake for the third dose of DTwP/PCV13 in Cameroon has been over 80% [16]. Prior to PCV13 introduction in July 2011 in Cameroon, no pneumococcal conjugate vaccination (neither PCV7 nor PCV10) were included in the national immunization programme.

Serotype-specific disease impact is important both to monitor existing programs and to guide design and implementation of planned future pneumococcal conjugate vaccines. However, few data exist from Cameroon on overall or serotype-specific PCV13 impact [15, 17]. The primary objective of this study was to describe trends in laboratory-confirmed pneumococcal meningitis by serotypes among PCV13-eligible children aged 2–59 months following introduction of PCV13 in Cameroon.

## 2. Methods

### 2.1 Ethical considerations

The Institutional Review Boards (IRBs) of the Cameroon Ministry of Scientific Research & Innovation and the Yaoundé Gynaecology, Obstetric and Paediatric Hospital approved the study. Parents verbally consented to their child’s participation in disease surveillance during routine medical care, for paediatric bacterial meningitis in Cameroon. Additionally, during specific follow up of cases identified via routine IPD surveillance, vaccine history and signed informed consent forms were obtained from the parents/caretakers of each participating child.

### 2.2 Study design, study setting and selection of study participants

We retrospectively analyzed laboratory-based surveillance data from August 2011 to December 2018. Additional information was obtained by review of patients’ medical reports for consented subjects. Retrieved demographic and clinical information were keyed into study-specific case report forms (CRFs). As previously described [5], study sites hosted at the mother and child hospital (MCH), a paediatric reference hospital in Yaoundé constitute a group of primary and secondary healthcare institutions (covering localities situated within 80 km radius from Yaoundé, Cameroon’s capital city) that were involved with the sentinel surveillance of invasive diseases. Yaoundé and its surroundings has a population of over 3.5 million inhabitants, of which 18% are children aged under-five years based on 2010 National Population Census [18]. The MCH is one of the largest children’s hospitals in the country and it is accessible and affordable to the local population. MCH keeps records of hospital visits, admissions, and deaths, in addition to specific clinical, laboratory and serotype data on invasive diseases including *Haemophilus influenzae* and *Streptococcus pneumoniae*. It has a capacity of 300 beds and records over 12000 admissions annually [5].

The study population included children aged from 2- to 59-months during the periodwho were admitted at any of the five surveillance hospitals within the *Surveillance Epidemiologique en l’Afrique Centrale (SURVAC)* network in Yaoundé, and who had cerebrospinal fluid (CSF) collected for suspected bacterial meningitis. Moreover, subject selection was based on any child meeting the inclusion criteria with a lumbar puncture performed and a CSF WBC result available. (However, only children in the 2 to 11month old age bracket are eligible to receive free vaccination in the country, although no catch-up vaccinations for the PCV13 were planned for infants above 4months old.) The SURVAC network was supported by the World Health Organization (WHO) country office in Cameroon and managed by the MCH/Ministry of Public Health. SURVAC was established as a routine infectious disease surveillance tool based on infections due to invasive pathogens identified as part of standard medical care from normally sterile sites (blood, pleural fluid, lung aspirates or CSF samples). IPD was added to the SURVAC network in August 2011.

### 2.3 Case definitions

The case definitions used in the surveillance hospitals for paediatric bacterial meningitis followed those of the WHO global invasive bacterial vaccine preventable diseases (WHO/IBVPD) guidelines [19]. However, in this study we had defined purulent meningitis as suspected meningitis with a CSF white blood cell (WBC) count ≥20 per mm^3^. Although WHO also includes elevated protein or decreased glucose as part of the case definition, but these tests were not routinely performed in Cameroon, as is true in much of West Africa. Patients with a CSF WBC count <20 per mm^3^ were not considered to have purulent meningitis (i.e. non-cases). A confirmed case of pneumococcal meningitis was defined as the identification of *S*. *pneumoniae* from CSF either by culture, antigen detection and/or PCR.

### 2.4 Data collection

Laboratory surveillance registers and patients’ files were reviewed and information extracted into study specific CRFs. Additional data were collected during parental/guardian outreach (N = 1923). Abstracted information included demographic data, date of admission, PCV vaccination history, clinical course and laboratory diagnoses.

### 2.5 Microbiological analysis of CSF specimens

As part of the SURVAC network, residual CSF collected during standard medical care from children hospitalized at the surveillance hospitals were assessed for appearance and WBC count, as previously reported [20]. With regards to culture, 10 microliters of CSF were inoculated onto gentamicin blood agar and chocolate agar plates, and plates were incubated overnight at 37°C in 5% carbon dioxide atmospheric conditions for 18 to 24 hours, as previously reported [21]. After overnight culture, plates were examined for the respective characteristic morphological growth of pathogens, including *S*. *pneumoniae*, *H*. *influenzae* and *N*. *meningitidis*. Otherwise, for CSF specimens from hospitalized children with suspected bacterial infection, a rapid antigen test was used, BinaxNOW^™^ for pneumococcal identification or PASTOREX^™^ for the other pathogens.

Colonies suspected to represent *S*. *pneumoniae* were confirmed by optochin susceptibility test (5μg optochin disc: Oxoid)with respect to the manufacturer’s instructions. All confirmed isolates were placed in 1ml STGG tubes containing transport media broth with 16% glycerol. After treatment in the STGG tubes, the confirmed isolates were stored at temperatures of -70°C, as were any CSF specimens where a confirmed isolate had not been obtained. Confirmed isolates and CSF specimens were shipped in dry ice (-70°C) for further testing and serotyping at the World Health Organization Reference Regional Laboratory (WHO/RRL), Medical Research Council (MRC), The Gambia (see Tables 4 and 5). During the first years (2011–2012)of SURVAC only culture or antigen positive specimens were sent to the WHO/RRL, but later all suspected specimens were shipped (Table 4).

### 2.6 Serotyping of pneumococcal isolates

At the WHO/RRL, real-time polymerase chain reaction (PCR) detection technique was used for serotyping. As earlier reported [21], RNAse P gene assay was applied on all CSF specimens to confirm that the samples were of human origin and to monitor the efficiency of amplification, which consisted of a series of cycles. First, there was an initial denaturation step at 95°C for 10 minutes, followed by 45 cycles of 95°C for 15 seconds and 60°C for 60 seconds. For each cycle of the targets, positivity was deduced using a cycle threshold (Ct) values, and Ct values of ≤36 were considered positive.

A CSF aliquot of 200 μl was added to 50 μl of TE buffer containing 0.08 g/ml of lysozyme (Sigma, L-6876) and 150 U/ml of mutanolysin (Sigma, M-9901), and the mixture was incubated for 1 hour at 37°C. Next, purified DNA extracts were exposed to a sequential triplex quantitative PCR (qPCR) assay designed to detect 21 pneumococcal capsular serotypes/serogroups using the African scheme as earlier reported [22]. Pneumococci with Ct values ≤32 by qPCR that could not be serotyped/serogrouped under this African scheme (Table 1) were serotyped by conventional multiplex serotyping PCR assays [23] (Serotype data for other pathogens were not available.).

**Table 1:** Pneumococcal capsular serotypes/serogroups primers used in the African schemes.

Reaction no.	Africa
1	1, 5, 23F
2	4, 6A/6B/6C/6D, 9V/9A
3	14, 18C/18A/18B/18F, 19F
4	3, 7F/7A, 19A
5	6C/6D, 12F/12A/12B/44/46, 22F/22A
6	15A/15F, 23A, 33F/33A/37
7	2, 11A/11D, 16F

Source: Adapted from Pimenta et al., 2013 [22]

### 2.7 Statistical analysis

We defined the years 2011-2014 as the early PCV13 era (EPE) and years 2015-2018 as the late PCV13 era (LPE). No surveillance data were available from the period before PCV13 introduction. Therefore, we performed descriptive analyses over time for serotype distribution among laboratory-confirmed pneumococcal and purulent meningitis cases. Serotypes were classified as vaccine types (VST: 1, 3, 4, 5, 6A, 6B, 7F, 9V, 14, 18C, 19A, 19F and 23F), non-vaccine types (NVST) and non-typeable pneumococci. For analytical purposes we have reported non-typeable serotypes as non-vaccine serotypes. We used the **χ**^2^-test to compare differences in the proportion of baseline characteristics among pneumococcal meningitis cases between the EPE and LPE. Analyses were conducted using theInternational Business Machines (IBM) corporation’s Statistical Package for Social Sciences (SPSS) version 25.0.

## 3. Results

### 3.1 Enrolment and baseline characteristics of participants

We enrolled 3131 children 2-59 months of age who had been evaluated for suspected meningitis and had a CSF collected. The median age of these children was 12 months (IQR: 6 to 24 months), 57.9% (1814/3131) were male, 45.6% were within the Cameroon NIP free vaccine-eligible age group of 2 to 11 months and 9.3% of the total number enrolled had a documented PCV13 vaccination history (Table 2).

**Table 2:** Demographics and vaccination status of children aged 2 – 59 months evaluated for suspected meningitis and admitted at the sentinel surveillance hospitals in Yaoundé: 2011—2018 (N = 3131).

Characteristics	Number	%
**Gender**		
Male	1814	57.9
Female	1317	42.1
**Age in months, median (IQR)**	12 (6 -24)	
**Age group (in months categorized)**		
2 to 11	1428	45.6
12 to 23	865	27.6
24 to 35	492	15.7
36 to 47	253	8.1
48 to 59	93	3
**Residence within Yaoundé**		
Yes	2821	90.1
No	310	9.9
**PCV13 Vaccination status**		
1 dose	67	2.1
2 doses	52	1.7
3 doses	172	5.5
Undocumented	2840	90.7

PCV13 = 13-valent pneumococcal conjugate vaccine; N = number; % = Percentage; IQR = Interquartile range.

CSF was clear on appearance for 67.3%, and 13.6% had received antibacterial medication before collection of CSF (Table 3).

**Table 3:** Clinical and laboratory outcomes of children aged 2 – 59 months evaluated for suspected meningitis admitted at the sentinel surveillance hospitals in Yaoundé: 2011—2018 (N = 3131).

Characteristics	Number	%
**Diagnosis at time of discharge**		
Bronchopneumonia	33	1.1
Pneumococcal meningitis (laboratory confirmed)	103	3.3
Septicaemia	83	2.7
Others	1283	40.9
Unknown (missing data)	1629	52.0
**Previous antibiotic use prior to admission**		
Yes, with/without medical prescription	426	13.6
No	2233	71.3
Unknown	472	15.1
**CSF appearance**		
Clear	2108	67.3
Turbid	281	9
Xanthrochromic	361	11.5
Others	381	12.2
**WBC counts in CSF (cells/mm**^**3**^**)**		
< 20	2865	91.5
20 to 99	168	5.4
≥ 100	98	3.1
**Outcome at discharge**		
Discharged to their homes alive	1331	42.5
Transferred alive to another hospital/Sequalae	18	0.6
Left against medical advice (alive)	88	2.8
Death	77	2.5
Undocumented	1617	51.6

WBC = white blood cells; CSF = Cerebrospinal fluid; mm^3^ = cubic milliliters, % = percentage.

**Fig 1:**
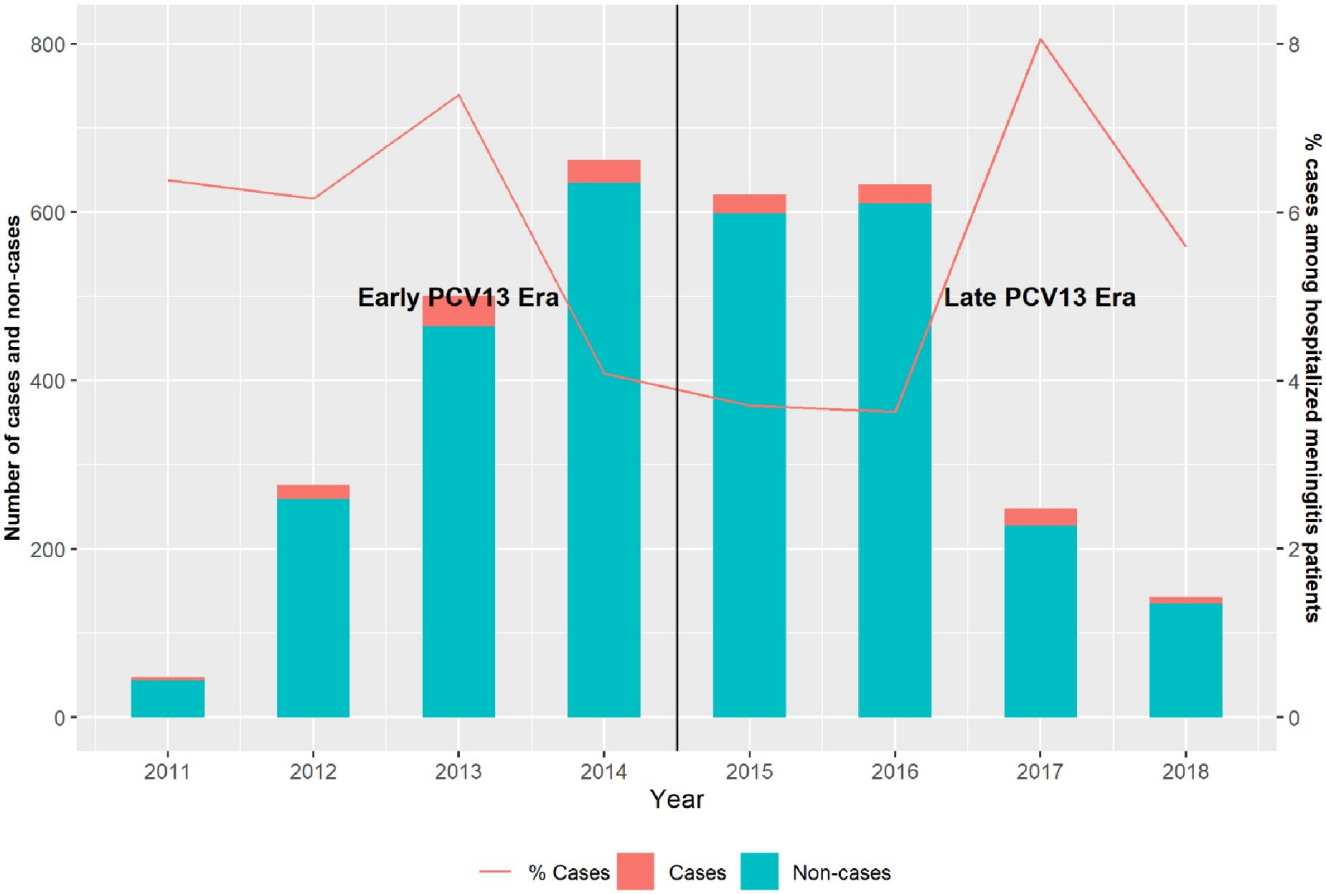
Profile of meningitis (purulent [Cases] and non-purulent [Non-cases]) in 2- to 59-month-old children hospitalized in Yaoundé, 2011 – 2018. Note: PCV13 = 13-valent pneumococcal conjugate vaccine; CSF = cerebrospinal fluid; WBC = white blood cell; Cases = Purulent meningitis patients with a CSF WBC count ≥20 per mm^3^; Non-cases = Meningitis patients with a CSF WBC count <20 per mm^3^.

The proportion of CSF specimens with purulence varied substantially by year with only modest and non-significant differences (p = 0.0846) between the EPE, 7.5% (112/1486) and the LPE, 9.4% (154/1645) (Fig 1).

### 3.2 Pathogen distribution

A representativesub-set of samples was chosen annually for the years between 2012 and 2018 (by contrast, in the partial year 2011, when PCV13 was introduced in July, all the available CSF samples were shipped to the WHO/RRL). A sub-set of CSF specimens (N = 765), were subsequently found to have been tested using either microbiological culture, antigen detection and/or polymerase chain reaction (PCR). Of this subset of 765 CSF specimens, 142 were laboratory confirmed as cases of bacterial meningitis including 103 resulting from S. *pneumoniae*, 13 from H. *influenzae* and 5 of *N*. *meningitidis* and 21 were a collection of other organisms. During every study year, pneumococcus was the most frequent pathogen identified by culture (Table 4).

**Table 4:** Pathogens identified by culture (all pathogens) or other methods (pneumococcus only) from cerebrospinal fluid specimens of children aged 2–59 months from Cameroon.

Year	*Streptococcus pneumoniae*			*Haemophilus influenzae*	*Neisseria meningitis*	Others	Sub-set of CSF specimen analyzed	Number of CSF specimens
	Culture	Antigen detection	PCR*	Culture	Culture	Culture		(CSF within Yaoundé)
**2011**	1	0	3	0	0	0	47	47 (18)
**2012**	5	0	5	3	2	4	62	276 (276)
**2013**	13	3	26	2	1	3	116	501 (473)
**2014**	8	2	16	3	1	4	154	662 (626)
**2015**	7	2	19	1	0	4	145	621 (563)
**2016**	6	1	15	4	0	5	149	633 (537)
**2017**	9	0	12	0	0	0	57	248 (207)
**2018**	6	0	7	0	1	1	35	143 (121)
**Total**	54	8	103	13	5	21	765	3131 (2821)

CSF = Cerebrospinal fluid; PCR = polymerase chain reaction; Others = (included *E*. *coli*, *Shigella*, *Salmonella* and *Klebsiella* species).

*Because PCR was used as the final and confirmatory method for pathogen identification, the total number of confirmed *S*. *pneumoniae* is the same as that of PCR.

### 3.3 *Streptococcus pneumoniae* serotypes

The WHO/RRL in The Gambia did not use Quellung but rather PCR for serotyping, which in some cases did not distinguish individual serotypes (for example, 18A/18B/18C/18F). As shown in Table 5, of the 103 children with pneumococcal meningitis evaluated for serotype, a vaccine serotype was present in 62% (31/50) of cases in the EPE versus 35.8% (19/53) in the LPE, showing a significant difference between the two periods (p = 0.0081).

**Table 5:** Vaccine-type and non-vaccine type serotype distribution among 2-59 months old children hospitalized at the sentinel surveillance hospitals with confirmed Pneumococcal Meningitis (PM) in Yaoundé: 2011—2018 (N = 103).

Serotypes^♫^/serogroups	Early PCV13 Era		Late PCV13 Era		PD (95%CI)	P-value
	[2011–2014], N = 50		[2015–2018], N = 53			
	n	%	n	%		
**Vaccine serotypes (VST)**						
**PCV7**						
**4**	2	4	3	5.6	1.6%[-8.6 to 11.7]	0.7062
**6A/6B**	12	24	0	0	-24.0% [-37.4 to 12.3]	**0.0002**
**9V**	0	0	0	0	0	0
**14**	2	4	7	13.2	9.2% [-2.4 to 21.2]	0.1000
**18C**	0	0	3	5.6	5.6% [-2.4 to 15.3]	0.0911
**18A/18B/18C/18F**	2	4	0	0	-4% [-13.5 to 3.4]	0.1434
**19F**	0	0	0	0	0	0
**23F**	1	2	0	0	-2% [-10.5to 4.9]	0.3032
**PCV10—PCV7**						
**1**	2	4	0	0	-4% [-13.5 to 3.4]	0.1434
**5**	3	6	2	3.8	-2.2% [-12.8 to 7.6]	0.6058
**7F/7A**	2	4	0	0	-4% [-13.5 to 3.4]	0.1434
**PCV13—PCV10**						
**3**	0	0	4	7.6	7.6% [-0.9 to 17.9]	**0.0478**
**6A**	3	6	0	0	-6% [-16.2 to 1.8]	0.0717
**19A**	2	4	0	0	-4% [-13.5 to 3.4]	0.1434
***VST sub-total***	***31***	***62***	***19***	***35*.*8***	-26.2% [-42.9 to 6.9]	***0*.*0081***
**Non-vaccine serotypes (NVST)**						
**PCV20—PCV13**						
**8**	0	0	3	5.6	5.6% [-2.4 to 15.3]	0.0911
**10A**	0	0	0	0	0	0
**11A/11D**	1	2	0	0	-2% [-10.5 to 4.9]	0.3032
**11A/11D/11F**	1	2	0	0	-2% [-10.5 to 4.9]	0.3032
**12F**	2	4	0	0	-4% [-13.5 to 3.4]	0.1434
**12F/12A/12B/44/46**	4	8	0	0	-8% [-18.8 to 0.32]	**0.0366**
**15B**	0	0	6	11.3	11.3% [1.9 to 22.6]	**0.0148**
**22F**	0	0	0	0	0	0
**33F**	0	0	0	0	0	0
**OTHERS**						
**2**	0	0	6	11.3	11.3% [1.9 to 22.6]	**0.0148**
**7C/7B/40**	0	0	2	3.8	3.8% [-3.8 to 12.8]	0.1660
**16F**	0	0	4	7.5	7.5% [-0.9 to 17.8]	**0.0494**
**17F**	0	0	2	3.8	3.8% [-3.8 to 12.8]	0.1660
**21**	0	0	2	3.8	3.8% [-3.8 to 12.8]	0.1660
**25F/25A/38**	2	4	0	0	-4% [-13.5 to 3.4]	0.1434
**33**	0	0	2	3.8	3.8% [-3.8 to 12.8]	0.1660
**35B**	5	10	0	0	-10% [-21.4 to 1.2]	**0.0188**
***NVST sub-total***	***15***	***30***	***27***	***51***	21.0% [2.0 to 37.9]	***0*.*0310***
**Non-typeable sub-total**	***4***	***8***	***7***	***13*.*2***	5.2% [-7.5 to 17.8]	*0*.*3953*
**TOTAL**	**50**	**100**	**53**	**100**		

PCV13 = 13-valent pneumococcal conjugate vaccine; N or n = number; % = percent; PCV20 = Non-PCV13 serotypes included in the new candidate vaccine serotypes + PCV13 serotypes; PD = Percentage difference; CI = Confidence interval; p-values in bold depict statistical significance differences in serotype distribution between the two periods; p-value estimates were computed using the Pearson Chi-Square Test; Bold font = depicts statistical significance differences in serotype distribution between the two periods. The serotypes that appear alone were obtained by polymerase chain reaction if the African scheme did not yield a serotype.

Also, during this period, there were modest and non-significant increases in the proportion of children with pneumococcal meningitis among older age categories and from outside Yaoundé. Serotype-specific distribution differed between the EPE and LPE, respectively (Table 6).

**Table 6:** Comparison of some epidemiological characteristics between the ’early and late post-PCV13 impact periods of 2–59 months old children with laboratory-confirmed pneumococcal meningitis hospitalized at the sentinel surveillance hospitals in Yaoundé: 2011—2018 (N = 103).

Characteristics	Early PCV13 Era	Late PCV13 Era	
	N (%)	N (%)	p-value
**Gender**			
Male	26 (52.0)	25 (47.2)	0.624
Female	24 (48.0)	28 (52.8)	
**Age in months, median (IQR)**	7 (3–13)	9 (3–18)	
**Age group (in months)**			
2 to 11	35 (70.0)	30 (56.6)	0.062
12 to 23	13 (26.0)	12 (22.6)	
24 to 35	2 (4.0)	6 (11.3)	
36 to 47	0 (0)	5 (9.4)	
48 to 59	0 (0)	0 (0)	
**Place of residence**			
Within Yaoundé	49 (98.0)	42 (79.2)	0.071
Outside Yaoundé	1 (2.0)	11 (20.8)	
**PCV13 serotype**			
Vaccine serotypes	31 (62.0)	19 (35.8)	0.014
Non-vaccine serotypes	19 (38.0)	34 (64.2)	

PCV13 = 13-valent pneumococcal conjugate vaccine; N = number; % = Percentage; p-value were computed using the Pearson’s Chi-square Test; IQR = Interquartile range.

## 4. Discussion

We evaluated changes in serotype causing pneumococcal meningitis during the early and later periods of PCV13 implementation in Cameroon. Our data suggest a substantial vaccine impact, with significantreductions in the proportion of pneumococcal meningitis due to VSTs and a shift in the specific serotypes causing meningitis. Specifically, the vaccine serotypes 1, 6A/B, 19A, and 23F were not identified by the end of the surveillance period. Similar trends in VST pneumococcal meningitis reduction have been reported previously in some Sub-Saharan African countries and elsewhere [10, 12, 24–30]. In The Gambia, PCV13 implementation in 2011 (following the switch from PCV7) resulted in reductions in invasive pneumococcal diseases [31]. This was as a result of a marked reduction in the proportion of PCV13-serotypes by 2014, with no cases in the two succeeding years (2015 and 2016) of pneumococcal meningitis isolated among children under-five within the surveillance system [25]. Similarly, the introduction of the PCV10 in Mozambique in 2013 led to rapid declines in the proportion of pneumococcal meningitis cases, down to 1.9% in 2015 from a 2013 baseline of 33.6%, among children less than five years old [10].

With respect to purulent meningitis, we did not see changes in the proportion of CSF samples with purulence in this study, although purulence can be due to multiple pathogens, not only *S*. *pneumoniae*. While this may reflect any substantial fluctuations in surveillance that occurred over the surveillance period, it could also suggest limitations of the PCV13 programme as implemented in Cameroon, either due to the accelerated schedule with no booster or possibly because of coverage levels that were less than those reported by the Ministry of Health. The latter may explain the frequent identification of PCV13 VSTs during the LPE. Other data from countries around the meningitis belt, which all use an accelerated infant schedule with no booster, and no catch-up at vaccine introduction, support this as well [11, 12, 26]. In Senegal, data from sentinel surveillance hospitals demonstrated a small, but significant reduction in all-cause pneumonia hospitalization 2 years following PCV13 infant vaccination, but similar trends were not noticeable in children aged 12–59 months or in meningitis cases [24].

In addition to ongoing disease due to VSTs, NVSTs arose during the LPE, such as from serotypes 8 and 15B. This may reflect trends in serotype-specific pneumococcal circulation related to confounding factors such as time since vaccination, varying population immunity, changes in antibiotic use patterns, or phenotypic shifts in organism invasiveness, as previously reported [29, 32]. Part of the shift also may be due to replacement of VSTs by NVSTs due to PCV13 effectiveness, a phenomenon which has been documented globally [27, 28, 33].

Unfortunately, Cameroon’s surveillance system did not allow calculation of incidence nor robust comparisons given the large changes in surveillance system robustness over time (Table 7).

**Table 7:** Some of the observed indicators/factors influencing surveillance programme performance in Cameroon.

**Supply of reagents**: Since the SURVAC project was not self-supported but relied entirely on stock supplies from partners, this was not usually timely and, in some cases, resulted in delays of the analyses. **Reporting and completion of entries**: Because electronic data is not maintained, tracking caregivers to get some clinical/vaccine history was problematic. Hence, this contributed to the missing data scenario. **Consistency**: The use of the international classification of diseases (ICD) codes is not mandatory in Cameroon, and this makes it really challenging to apply standard case definitions on clinical diagnosis.

Other substantial study limitations existed. The over 80% DTwP/PCV13 vaccination coverage reported in the text are administrative figures [16]. It is likely that most children have been immunized, even with an undocumented vaccination history, through bi-annual mass vaccination campaigns or supplementary immunization activities. In contrast to the DTwP and PCV13 vaccination coverage administrative figures, however, PCV13 vaccination history in this study was available only on 9.3% of subjects due to the retrospective nature of the effort to obtain that information. Children were identified with documented evidence of PCV13 vaccination through contact tracing, either by specific follow-up of vaccination status for cases that had been identified through routine IPD surveillance or by review of patients’ medical records. Another additional limitation of the PCV13 programme implemented in Cameroon is that it relies on an accelerated primary vaccination schedule with no booster. Further, sentinel surveillance was only initiated at the time of vaccine introduction, so data were not available from the pre-vaccination era, though this was undertaken in other Sub-Saharan countries [11, 12, 26]. Furthermore, in our study pathogen identification was not done routinely in a systematic fashion, which limited our ability to account appropriately for the sensitivity of each diagnostic method. We had considered this as a potential source of bias to our findings, but as documented with the prevalence of pneumococcal carriage in the era of conjugate vaccines, our data is consistent with the trends of decrease in some VST accompanied with a simultaneous increase in some NVST [34–36]. Pneumococcal carriage has been reported to be a precursor to invasive pneumococcal diseases (including pneumococcal meningitis), and the changes seen in carriage are most likely to be seen in disease [37]. A previous study conducted on carriage with infants from this population four years after PCV13 introduction had obtained similar trends [17]. However, as outlined in Table 4, because PCR was used for pathogen confirmation and serotyping, we used the total number of confirmed *S*. *pneumoniae* result as obtained by the PCR technique that has generally been used for molecular characterization [11, 12, 26].

Surveillance was conducted only in the Yaoundé area, potentially limiting generalizability of the findings. Burkina Faso for example, performs nation-wide meningitis surveillance and routine serotyping of all pneumococcal meningitis specimens. By contrast, only sentinel surveillance is performed in Cameroon since its inception. Specimens were not serotyped locally and only a sub-set of specimens shipped to the WHO regional reference laboratory at the MRC in The Gambia for serotyping which may have resulted in a low proportion of pneumococcal serotypes being identified. Also, missing data may have impacted representativeness of our findings. However, since most children in the study, and in Cameroon, are living under the poverty line [18], their risk factors are likely broadly similar for disease outcomes and vaccination.

Despite these limitations, these are the first data following PCV13 introduction in Cameroon and thus provide information to help guide future decision-making. For example, given ongoing PCV13 VST circulation, Cameroon should assess true coverage—including by number of doses per infant—through surveys, and should confirm the strength of the vaccine cold-chain. The possible shift of cases to ages outside of infancy, and ongoing PCV13 VST circulation, adds to recent recommendations that moving to a 2+1 schedule may be a more effective three-dose PCV schedule [12]. The common identification of serotypes contained in one of the current candidates PCVs (PCV20) suggests that expanded valency vaccines may be of substantial benefit. Lastly, decision-making in Cameroon would be greatly enhanced by building and strengthening a consistent case-based IPD (including meningitis, pneumonia and septicemia) surveillance system.

## Supporting information

S1 Dataset (SAV)
