# Correction: Impact of 13-valent pneumococcal conjugate vaccine on laboratory-confirmed pneumococcal meningitis and purulent meningitis among children ˂5 years in Cameroon, 2011–2018

**DOI:** 10.1371/journal.pone.0254616

**Published:** 2021-07-09

**Authors:** The PLOS ONE Staff

The second author’s name is listed incorrectly. The correction citation is: Njuma Libwea J, Fletcher MA, Koki Ndombo P, Boula A, Ashukem NT, Ngo Baleba M, et al. (2021) Impact of 13-valent pneumococcal conjugate vaccine on laboratory-confirmed pneumococcal meningitis and purulent meningitis among children ˂5 years in Cameroon, 2011–2018. PLoS ONE 16(4): e0250010. https://doi.org/10.1371/journal.pone.0250010. The sixteenth author’s name is spelled incorrectly. The correct name is: Jo Southern. The publisher apologizes for these errors.
