# Supplementary material for: Impact of 13-valent pneumococcal conjugate vaccine on laboratory-confirmed pneumococcal meningitis and purulent meningitis among children ˂5 years in Cameroon, 2011–2018
Source: PLoS One. 2021 Apr 15;16(4):e0250010. doi: 10.1371/journal.pone.0250010 (PMC8049353; doi:10.1371/journal.pone.0250010)
Supplement: Attachment — Submitted filename: Revisione .docx [file pone.0250010.s002.docx]

**Impact of 13-valent pneumococcal conjugate vaccine in Cameroon on laboratory-confirmed pneumococcal meningitis and purulent meningitis among children < 5 years, 2011-2018**

The authors conducted a retrospective observational study in patients aged< 5 yrs hospitalized for clinically suspected meningitis (CSBM,) admitted to hospitals in Yaoundè, capitol city of Cameroon, from August 2011 to December 2018. They declared aim of this study was to describe the impact of PCV13 on serotype distribution among laboratory confirmed pneumococcal meningitis cases over this period of time.

**General comment**

The authors report well known published information on pneumococcal meningitis from neighboring countries of the African meningitis belt, before and after the introduction of PCV-13. However, their article presents many shortcomings in data collection, description of laboratory testing, statistical analyses and results.

**Major comments**

- The study evaluated children born before the introduction of PCV-13 and does not specify which children had been vaccinated with PCV-13 and which one had received PCV-7.

Authors declare that in Cameroon, since 2012, uptake for the third dose of PCV-13has been over 80%. However, in their study it is not emphasized that only 9.7% of children had a vaccination history. This approach offers a fundamental bias for evaluating objectively the effect of a correct immunization program on the epidemiology of meningitis.

- The authors mentioned that microbiological identification was obtained through bacterial culture, antigen testing and PCR. Unfortunately, incomplete information on the number of test performed and their sensitivity was not reported.

Methods

- Please provide some information regarding the study site (size of hospitals, type of hospitals (primary/secondary/tertiary care, pediatric care, for what population they are a referral center, etc..)
- The authors assert to have evaluated the “epidemiology” of pneumococcal meningitis during periods of PCV13 implementation in Cameroon. However, data are available only for 103 patients.

Did the authors calculate the power of the study and a sample size? The missing data may affect the generalizability of results.

Results

- The data about purulent meningitis is not clear. The authors defined purulent meningitis as “suspected meningitis with a CSF white blood cell (WBC) count ≥ 20 per mm3”. In the results the percentage of EEP and LPP is calculated on a total of 3131 purulent meningitis (1486 + 1645) but in Table 1b only 266 cases meet the criteria. Can the authors explain better the eligibility criteria and the selection of study participants?
- It would be better, if possible, that authors provide characteristics of the 103 study participants (e.g., demographic, age, …)
- It is appropriate to summarize asymmetrical distribution (as data in table 1a, table 1 b, table 3, table 4) by giving the median and percentile range (for example, 25th and 75th percentiles, giving the mean and standard deviation for continuous variable and to indicate the missing data for each variable.
- Table 1 b: the title describes the “characteristics aged 2-59 months evaluated for suspected meningitis”. In the table the number of suspected meningitis is 3026.
- Table 3: the title quotes a total of 105 participants (N = 105), actual total of results is 103.
- Reference 13 is not appropriate at the point of the discussion.

Discussion

- It is recommended to discuss in more detail the limitations of the study, taking into account sources of potential bias or shortcomings (study size, confounders, etc) and provide a conservative overall interpretation of data.
- Authors may compare the study being presented with other studies in the literature in terms of validity, generalizability, and precision.

Despite the laudable effort of the AA in collecting data, the study has not the power to add significant evidence on Pneumococcal meningitis trends in the contest of PCV-13 and to support a change in the vaccination schedule and in the understanding of Pneumococcal Meningitis epidemiology in Cameroon due the above expressed limits.
