# Supplementary material for: Impact of 13-valent pneumococcal conjugate vaccine on laboratory-confirmed pneumococcal meningitis and purulent meningitis among children ˂5 years in Cameroon, 2011–2018
Source: PLoS One. 2021 Apr 15;16(4):e0250010. doi: 10.1371/journal.pone.0250010 (PMC8049353; doi:10.1371/journal.pone.0250010)
Supplement: Attachment — Submitted filename: Response to Reviewers_PLoS One _ 4 Dec 2020_final.docx [file pone.0250010.s003.docx]

**Impact of 13-valent pneumococcal conjugate vaccine inCameroon on laboratory-confirmed pneumococcal meningitisand purulent meningitis among children < 5 years, 2011-2018**

The authors conducted a retrospective observational study in patients aged <5yrs hospitalized for clinically suspected meningitis (CSBM,) admitted to hospitals in Yaoundé, capitol city of Cameroon, from August 2011 to December 2018. They declared aim of this study was to describe the impact of PCV13 on serotype distribution among laboratory confirmed pneumococcal meningitis cases over this period.

**General comment**

The authors report well known published information on pneumococcal meningitis from neighboring countries of the African meningitis belt, before and after the introduction of PCV-13. However,their article presents many shortcomings in data collection, description of laboratory testing, statistical analyses, and results.

_____________________________________________________________________________

Dear Reviewer, we recognize the limitations in our data, but nevertheless think that they are robust for the analyzed outcomes. Additionally, given the paucity of data on PCV impact from West Africa, with some of the highest pneumococcal disease burdens globally, we think our results are an important contribution to the literature.

**Major comments**

**Comment No. 1:**The study evaluated children born before the introduction of PCV-13 and does not specify which children had been vaccinated with PCV-13 and which one had received PCV-7.

Response No. 1:The highlighted following text has now been included in the 4th paragraph, lines 8 to 10 in the background section to provide clarity: "Prior to PCV13 introduction in July 2011 in Cameroon, no pneumococcal conjugate vaccination (neither PCV7 nor PCV10) was included in the national immunization programme". In addition, in this study we evaluated only children born from August 2011 onwards who were, therefore, all eligible for PCV13 vaccination.

**Comment No. 2:**Authors declare that in Cameroon, since 2012, uptake for the third dose of PCV-13 has been over 80%.However, in their study it is not emphasized that only 9.7% of children had a vaccination history. This approach offers a fundamental bias for evaluating objectively the effect of a correct immunization program on the epidemiology of meningitis.

Response No. 2: We thank the reviewer for this observation. The following statement on this has now been included (Results section, 1st paragraph line 4): “…and 9.3% of the total number enrolled had a documented PCV13 vaccination history”.

We have also added additional text in the Discussion (page 15, see 4^th^ paragraph, lines 3 to 8): "The lack of robust vaccination history means that fewer than 80% of children may have been vaccinated, potentially explaining why more dramatic reductions weren't seen.It may also suggest limitations of the PCV13 programme as implemented in Cameroon, either due to accelerated primary vaccination schedule with no booster or possibly because of vaccine uptake levels that were less than those reported by the administration. The latter would explain the frequent identification of PCV13 VSTs during the LPE.”

**Comment No. 3:**The authors mentioned that microbiological identification was obtained through bacterial culture, antigen testing and PCR. Unfortunately, incomplete information on the number of tests performed and their sensitivity was not reported.

Response No. 3: We have provided additional information on why this was so. The following sentence has been included in section 2.5, lines 7-8: "During the early years of SURVAC, only culture or antigen positive specimens were sent to the WHO/RRL but later all suspected specimens were shipped (Table 2)". Also, we have updated the results section 3.2 with corresponding information to support the ensuing statement from section 2.5: Only a sub-set of CSF specimens (N=765), were subsequently found to have been tested using either microbiological culture, antigen detection and/or polymerase chain reaction (PCR). Of this subset of 765 CSF specimens, 142 were laboratory confirmed as cases of bacterial meningitis including 103 resulting from S. *pneumoniae*, 13 from H*. influenzae*, 5 of *N. meningitidis* and 21 were a collection of other organisms.

Methods

**Comment No. 4:**Please provide some information regarding the study site (size of hospitals, type of hospitals (primary/secondary/tertiary care, pediatric care, for what population they are a referral center, etc..).

Response No. 4:

The following description has been added to the manuscript (see section “2.2 Study design, setting and selection of study participants”, paragraph 1, lines 4 to 13): "As previously described [5], study sites hosted at the paediatric reference hospital in Yaounde (MCH) constitute a group of primary and secondary healthcare institutions (covering localities situated within 80 km radius from Yaoundé, Cameroon's capital city) that were involved with the sentinel surveillance of invasive diseases. Yaoundé and its surroundings have a population of over 3.5 million, of which 18% were children aged under-five years based on 2010 National Population Census. The MCH is one of the largest children’s hospitals in the country and it is accessible and affordable to the local population. MCH keeps records of hospital visits, admissions, and deaths, in addition to specific clinical, laboratory and serotype data on invasive diseases including Haemophilus influenzae and Streptococcus pneumoniae. It has a capacity of 300 beds and records over 12000 admissions annually".

**Comment No. 5:**The authors assert to have evaluated the “epidemiology” of pneumococcal meningitis during periods of PCV13 implementation in Cameroon. However, data are available only for 103 patients.

Response No. 5:

We thank the reviewer again for this observation and acknowledge that the data available only for 103 children with a pneumococcal serotype may be sparse, but this could be explained when considering the surveillance programme limitations that are presented in the manuscript discussion. Also, we explained in section 2.5, lines 7 and 8 that, during the early years of SURVAC only culture or antigen positive specimens were sent to the WHO/RRL but later all suspected specimens were shipped. The text has been updated, so that the term epidemiology is removed from the first line of the discussion: "We evaluated changes in the distribution of serotypes causing penumococcal meningitis during the early and later periods of PCV13 implementation in Cameroon".( see Discussion section, paragraph 1, lines 1 to 2)

**Comment No. 6:**Did the authors calculate the power of the study and a sample size? The missing data may affect the generalizability of results.

Response No. 6: Yes, we had estimations for sample size and power of the study in the statistical analysis plan because the initial goal was to conduct a study with both retrospective and prospective arms. But due to logistical challenges, the prospective arm was not feasible at the time. Therefore, since this study involved just the retrospective arm that used register-based laboratory data, the entire retrospective database was used and as this was a descriptive study, it made sample size calculations unnecessary.

Results

**Comment No. 7:**The data about purulent meningitis is not clear. The authors defined purulent meningitis as “suspected meningitis with a CSF white blood cell (WBC) count ≥ 20 per mm3”. In the results the percentage of EEP and LPP is calculated on a total of 3131 purulent meningitis (1486 + 1645) but in Table 1b only 266 cases meet the criteria. Can the authors explain better the eligibility criteria and the selection of study participants?

Response No. 7: We thank the reviewer again for drawing our attention to this.

The study population included children aged from 2 to 59 months during the period (August 2011 to December 2018) who were admitted at any of the five surveillance hospitals within the *Surveillance Epidemiologique en l'Afrique Central* (SURVAC) network in Yaounde, and who had cerebrospinal fluid (CSF) collected for suspected bacterial meningitis (see section 2.2, second paragraph, lines 14 to 18).

Moreover, subject selection was based on any child meeting the inclusion criteria with a lumbar puncture performed and a CSF WBC result available.

The correct definition of purulent meningitis was included, “suspected meningitis with a CSF white blood cell (WBC) count ≥ 20 per mm3”. However, we had omitted to define in the text what was not considered as a purulent meningitis case (i.e. non-cases). This has now been added to the inclusion criteria text in the manuscript, section "2.3 Case definition": "Patients with a CSF WBC count <20 per mm^3^ were not considered to have purulent meningitis" (see section 2.3, paragraph 1 and line 4).

**Comment No. 8:**It would be better, if possible, that authors provide characteristics of the 103 study participants (e.g., demographic, age, …)

Response No. 8: This is provided for gender, age group, and place of residence (See Table 4).

If the Editors or Reviewer have additional items they would like to include, please let us know, although we may have limited ability to access these data.

**Comment No. 9:**It is appropriate to summarize asymmetrical distribution (as data in table 1a, table 1 b, table 3, table 4) by giving the median and percentile range (for example, 25th and 75th percentiles, giving the mean and standard deviation for continuous variable and to indicate the missing data for each variable.

Response No. 9: We agree with the Reviewer on the appropriateness of presenting asymmetric data by giving median and percentile range, but with mean and standard deviation for continuous variables (Categorizing the continuous variables {e.g. age, WBC counts}, as we have done, was to facilitate comparison with previous studies).

This has been updated were appropriate in the tables specified by the Reviewer. With respect to the missing data, this has been documented in Table 1a for "PCV13 vaccination status" ("Undocumented") and Table 1b for "Diagnosis at time discharge" ("Unknown {missing data}"), "Previous antibiotic use prior to admission" ("Unknown"), "CSF appearance" ("Others") and "Outcome at discharge" ("Undocumented").

**Comment No. 10:**Table 1 b: the title describes the “characteristics aged 2-59 months evaluated for suspected meningitis”. In the table the number of suspected meningitis is 3026.

Response No. 10: We thank the Reviewer for drawing our attention to this. Our intention was to describe the situation at the time of patient discharge from the hospital. This has now been modified in the manuscript, and the title reads "**Clinical and laboratory outcomes of children aged 2 – 59 months evaluated for suspected meningitis admitted at the sentinel surveillance hospitals in Yaoundé: 2011 ─ 2018 (N = 3131)",** and the category "Suspected meningitis" has been corrected to read "Diagnoses at time of discharge"

We also thank the Reviewer for pointing out the difference in the number of suspected meningitis cases. There were 3026 cases identified at the time of triage, as compared to 3131 cases with a discharge diagnosis, which was the outcome used for this study.

**Comment No. 11:**Table 3: the title quotes a totalof 105 participants (N = 105), actual total of results is 103.

Response No. 11: There was a typographical error that has now been rectified. The total number of participants now reads, N=103

**Comment No. 12:**Reference 13 is not appropriate at the point of the discussion.

Response No. 12: The reference has been removed, and the reference has been renumbered.

Discussion

**Comment No. 13:**It is recommended to discuss in more detail the limitations of the study,considering sources of potential bias or shortcomings (study size, confounders, etc) and provide a conservative overall interpretation of data. Authors may compare the study being presented with other studies in the literature in terms of validity, generalizability, and precision.

Response No. 13: This has been revised across the section. For instance, in the second paragraph under discussion, lines 1 -6; the sentence now reads "Despite these gains, we did not see changes in the proportion of CSF samples with purulence in our study. While this may reflect any substantial fluctuations in surveillance that occurred over the surveillance period, it could also suggest limitations of the PCV13 programme as implemented in Cameroon, either due to the accelerated schedule with no booster or possibly because of coverage levels that were less than those reported by the Ministry of Health. The latter may explain the frequent identification of PCV13 VSTs during the LPE."

There are additional discussion on the limitations on the third paragraph from line 4 to 16, as follows: "Sentinel surveillance was only initiated at the time of vaccine introduction, so data were not available from the pre-vaccination era, though this was undertaken in other Sub-Saharan countries [11,12,22]. Furthermore, in our study, pathogen identification was not done routinely in a systematic fashion, which limited our ability to account appropriately for the sensitivity of each diagnostic method. However, as outlined in Table 2, because PCR was used for pathogen identification and serotyping, we used the total number of confirmed *S. pneumoniae* result as obtained by the PCR technique that has generally been used for molecular characterization[11,12,22]. Surveillance was conducted only in the Yaounde area, potentially limiting generalizability of the findings. Burkina Faso for example, performs nation-wide meningitis surveillance and routine serotyping of all pneumococcal meningitis specimens. By contrast, in Cameroon during the early years of the surveillance programme, only sentinel surveillance was performed. Specimens were not serotyped locally and only culture-positive pneumococcal specimens were shipped to the WHO regional reference laboratory at the MRC in The Gambia for serotyping which may have resulted in a low proportion of pneumococcal serotypes being identified".
