# Supplementary material for: Impact of 13-valent pneumococcal conjugate vaccine on laboratory-confirmed pneumococcal meningitis and purulent meningitis among children ˂5 years in Cameroon, 2011–2018
Source: PLoS One. 2021 Apr 15;16(4):e0250010. doi: 10.1371/journal.pone.0250010 (PMC8049353; doi:10.1371/journal.pone.0250010)
Supplement: Attachment — Submitted filename: Response to Reviewers_PONE-D-20-24718R1_PLoS_Libwea_05MAR21.docx [file pone.0250010.s004.docx]

PONE-D-20-24718R1
Impact of 13-valent pneumococcal conjugate vaccine on laboratory-confirmed pneumococcal meningitis and purulent meningitis among children ˂ 5 years in Cameroon, 2011 - 2018
PLOS ONE

Dear Dr.John Njuma Libwea, 

Thank you for submitting your manuscript to PLOS ONE. After careful consideration, we feel that it has merit but does not fully meet PLOS ONE’s publication criteria as it currently stands. Therefore, we invite you to submit a revised version of the manuscript that addresses the points raised during the review process.

Please submit your revised manuscript by Mar 07 2021 11:59PM. If you will need more time than this to complete your revisions, please reply to this message or contact the journal office at [plosone@plos.org](mailto:plosone@plos.org). When you're ready to submit your revision, log on to [https://www.editorialmanager.com/pone/](https://urldefense.proofpoint.com/v2/url?u=https-3A__www.editorialmanager.com_pone_&d=DwQFaQ&c=UE1eNsedaKncO0Yl_u8bfw&r=FLGIKPNY_xSYZqyTXUJQEH0v1CsK33K-64KyoFi5s0A&m=HgDvybLMzCrDPQckl8JNR42feJOxCusWg4Slw5z1CH4&s=71m4u_xgspOfxD2v7JErcJCW1dpnh7mL3QcfysJxMTU&e=) and select the 'Submissions Needing Revision' folder to locate your manuscript file.

Please include the following items when submitting your revised manuscript:

- A rebuttal letter that responds to each point raised by the academic editor and reviewer(s). You should upload this letter as a separate file labeled 'Response to Reviewers'.
- A marked-up copy of your manuscript that highlights changes made to the original version. You should upload this as a separate file labeled 'Revised Manuscript with Track Changes'.
- An unmarked version of your revised paper without tracked changes. You should upload this as a separate file labeled 'Manuscript'.

If you would like to make changes to your financial disclosure, please include your updated statement in your cover letter. Guidelines for resubmitting your figure files are available below the reviewer comments at the end of this letter.

If applicable, we recommend that you deposit your laboratory protocols in protocols.io to enhance the reproducibility of your results. Protocols.io assigns your protocol its own identifier (DOI) so that it can be cited independently in the future. For instructions see: [http://journals.plos.org/plosone/s/submission-guidelines#loc-laboratory-protocols](https://urldefense.proofpoint.com/v2/url?u=http-3A__journals.plos.org_plosone_s_submission-2Dguidelines-23loc-2Dlaboratory-2Dprotocols&d=DwMFaQ&c=UE1eNsedaKncO0Yl_u8bfw&r=FLGIKPNY_xSYZqyTXUJQEH0v1CsK33K-64KyoFi5s0A&m=HgDvybLMzCrDPQckl8JNR42feJOxCusWg4Slw5z1CH4&s=a43iTw9avlAcHiQGN1Jwawyt9zvLe0HH8_RkdumGQmo&e=)

We look forward to receiving your revised manuscript.

Kind regards,

Silvia Ricci
Academic Editor
PLOS ONE

**Review Comments to the Author.**

Please use the space provided to explain your answers to the questions above. You may also include additional comments for the author, including concerns about dual publication, research ethics, or publication ethics. (Please upload your review as an attachment if it exceeds 20,000 characters)

Reviewer #2. There are three main issues that must be clarified by the authors: Without clearly describing these issues it is impossible to know the representativeness of the sample and consequently, the value of the results obtained.

Response: Dear Reviewer, we recognize the limitations in our data, but nevertheless think that they are robust for the analyzed outcomes. Additionally, given the paucity of data on PCV impact from West Africa, with some of the highest pneumococcal disease burdens globally, we think our results are an important contribution to the literature.

**Major comments**

Comment #1. The samples studied and their distribution by year must be clear. It is impossible to know how many CSF samples of those obtained were studied each year, by what technique and their results. How many samples were from Yaounde or from outside each year of the study. This must be clearly depicted in Table 2.

Response # 1: We thank the Reviewer for this observation. Distinguishing the data between all samples and those from Yaoundéhas been provided in Table 2 under the column of "Number of CSF samples (no. of CSF within Yaoundé)”. [See table at end of this Major Comments section.]

In 2011, for example, 18 of the 47 CSF samples were collected from children living within Yaoundé. PCR was used as the final and confirmatory method for pathogen identification, the total number of confirmed *S. pneumoniae* is the same as that of PCR. Consequently, in this same year, pathogens were identified from 3 of 47 CSF samples, which were all *S. pneumoniae*: 1 pathogen was isolated via culture with PCR confirmationand 2 were identified by PCR only.

Comment #2. Microbiological methods must be clearly described: what media were used for culture; what identification techniques were used, what antigen, what real-time PCR…

Response # 2: We do agree again with the Reviewer on the need to clearly demonstrate the aspect of reproducibility in scientific research in our methods. The following text hasbeen added under section 2.5.

**Microbiological analysis of CSF specimens**:

With regards to culture, 10 microliters of CSF were inoculated onto gentamicin blood agar and chocolate agar plates, and plates were incubated overnight at 37^0^C in 5% carbon dioxide atmospheric conditions for 18 to 24 hours, as previously reported (Boula et al., 2019; Kwambana-Adams et al., 2016). After overnight culture, plates were examined for the respective characteristic morphological growth of pathogens, including *S. pneumoniae*, *H. influenzae* and *N. meningitidis*. Otherwise, for CSF specimens from hospitalized children with suspected bacterial infection, a rapid antigen test was used, BinaxNOW^TM^ for pneumococcal identification or Pastorex for the other pathogens.

Colonies suspected to represent *S. pneumoniae* were confirmed by optochin susceptibility test (5µg optochin disc: Oxoid)with respect to the manufacturer's instructions. All confirmed isolates were placed in 1ml STGG tubes containing transport mediabroth with 16% glycerol. After treatment in the STGG tubes, the confirmed isolates were stored at temperatures of -70^0^C, as were any CSF specimens where a confirmed isolate had not been obtained.Confirmed isolates and CSF specimens were shipped in dry ice (-70^0^C) for further testing and serotyping at the World Health Organization Reference Regional Laboratory (WHO/RRL), Medical Research Council (MRC), The Gambia.

**Serotyping of pneumococcal isolates:**

At the WHO/RRL, real-time polymerase chain reaction (PCR) detection technique was used for serotyping. As earlier reported (Kwambana-Adams et al., 2016), RNAse P gene assay was applied on all CSF specimens to confirm that the samples were of human origin and to monitor the

efficiency of amplification,which consisted of a series of cycles. First, there was an initial denaturation step at 95^0^C for 10 minutes, followed by 45 cycles of 95^0^C for 15 seconds and 60^0^C for 60seconds. For each cycle of the targets, positivity was deduced using a cycle threshold (Ct) values, and Ct values of ≤36 were considered positive.

A CSF aliquot of 200µlwasadded to 50µl of TE buffer containing 0.08g/ml of lysozyme (Sigma, L-6876) and 150 U/ml of mutanolysin (Sigma, M-9901), and the mixture was incubated for 1 hour at 37^0^C. Next, purified DNA extracts were exposed to asequential triplex quantitative PCR (qPCR) assay designed to detect 21 pneumococcal capsular serotypes/serogroups using the African scheme as earlier reported (Pimenta et al., 2013). Pneumococci with Ct values ≤32 by qPCR that could not be serotyped/serogrouped under this African scheme were serotyped byconventional multiplex serotyping PCR assays. (Serotype data for other pathogens were not available.)

Comment #3. How is it possible that “since 2012, uptake for the third dose of DTwP/PCV13 in Cameroon has been over 80% [16]” and in the study only 9.3% of the total number enrolled had a documented PCV13 vaccination history (5.5% with 3 doses)?

Response # 3: We again agree with the Reviewer that this might be a point of concern. We had taken accountof these limitations in the Discussion section.

Challenges with missing data (e.g., undocumented vaccination history) are a phenomenon in most of resource-limited countries, and electronic vaccination records are scarce.We were unable to capture vaccination data because most children(90.7%) had an undocumented vaccination status due to parents reporting to the hospital without vaccination cards/bookletswhen their children were sick.

The over 80% DTwP/PCV13 vaccination coverage reported in the text are administrative figures[16]. It is likely that most children have been immunized, even with an undocumented vaccination history, through bi-annual mass vaccination campaigns or supplementary immunization activities. In contrast to the DTwP and PCV13 vaccination coverage administrative figures, however, PCV13 vaccination history in this study was available only on 9.3% of subjects due to the retrospective nature of the effort to obtain that information. Children were identified with documented evidence of PCV13 vaccination through contact tracing, either by specific follow-up of vaccination status for cases that had been identified through routine IPD surveillance or by review of patients' medical records.

| **Table 2. Pathogens identified by culture (all pathogens) or other methods (pneumococcus only) from cerebrospinal fluid specimens of children aged 2─59 months from Cameroon.** | | | | | | | | |
| --- | --- | --- | --- | --- | --- | --- | --- | --- |
| **Year** | ***Streptococcus pneumoniae*** | | | ***Haemophilus influenzae*** | ***Neisseria meningitis*** | **Others** | **Sub-set of CSF specimen analyzed** | **Number of CSF specimens(no. of CSF within Yaoundé)** |
|  | **Culture** | **Antigen detection** | **PCR*** | **Culture** | **Culture** | **Culture** |  |  |
| **2011** | **1** | **0** | **3** | **0** | **0** | **0** | **47** | **47 (18)** |
| **2012** | **5** | **0** | **5** | **3** | **2** | **4** | **62** | **276 (276)** |
| **2013** | **13** | **3** | **26** | **2** | **1** | **3** | **116** | **501 (473)** |
| **2014** | **8** | **2** | **16** | **3** | **1** | **4** | **154** | **662 (626)** |
| **2015** | **7** | **2** | **19** | **1** | **0** | **4** | **145** | **621 (563)** |
| **2016** | **6** | **1** | **15** | **4** | **0** | **5** | **149** | **633 (537)** |
| **2017** | **9** | **0** | **12** | **0** | **0** | **0** | **57** | **248 (207)** |
| **2018** | **6** | **0** | **7** | **0** | **1** | **1** | **35** | **143 (121)** |
| **Total** | **54** | **8** | **103** | **13** | **5** | **21** | **765** | **3131 (2821)** |
| CSF = Cerebrospinal fluid; PCR = polymerase chain reaction; Others = (included E. *coli, Shigella, Salmonella and Klebsiella species)**Because PCR was used as the final and confirmatory method for pathogen identification, the total number of confirmed S. pneumoniae is the same as that of PCR | | | | | | | | |

**Fig 1. Profile of meningitis(purulent [Cases] and non-purulent [Non-cases]) in 2- to 59-month-old children hospitalized in Yaoundé, 2011 - 2018.**

*Note: PCV13= 13-valent pneumococcal conjugate vaccine; CSF = cerebrospinal fluid; WBC = white blood cell; Cases = Purulent meningitis patients with a CSF WBC count ≥20 per mm^3^; Non-cases = Meningitis patients with a CSF WBC count <20 per mm^3^.*

**Other comments:**

Comment #1. “We retrospectively analyzed laboratory-based surveillance data from August 2011 to December 2018.” However, Fig 1. represents data from 2011 to 2019. What happened in 2019? There were no cases of pneumococcal meningitis? It would be desirable to have the incidence data of pneumococcal meningitis of both periods studied, to see if the introduction of vaccine has represented a decrease in the incidence of meningitis or not.

Response # 1:We had partial data, but no serotyping of the CSF specimens, for the first quarter of 2019, so this was not reported. It was an error to have uploaded partial 2019 values in the Figure 1; however, this has been rectified and now reads Fig 1. represents data from 2011 to 2018.

We agree with the Reviewer on the need to have incidence data. We initially aimedto estimate incidence;however, the surveillance system was not population based and the completeness of event ascertainment could have varied from year to year.The lack of incidence data truly limited the extend of the analyses that we had planned originally.Likewise,many studies, even in developed countries, report data on proportions rather than incidence because of the difficulty in obtaining consistent data from a well-definedpopulation catchment area.

To address the Reviewer's concern, in the Discussion, we emphasized the need for improved surveillance to be able to document further the utility of PCV13 immunization in Cameroon. The over 80% DTwP/PCV13 vaccination coverage reported in the text are administrative figures [16]. It is likely that most children have been immunized, even with an undocumented vaccination history, through bi-annual mass vaccination campaigns or supplementary immunization activities. In contrast to the DTwP and PCV13 vaccination coverage administrative figures, however, PCV13 vaccination history in this study was available only on 9.3% of subjects due to the retrospective nature of the effort to obtain that information. Children were identified with documented evidence of PCV13 vaccination through contact tracing, either by specific follow-up of vaccination status for cases that had been identified through routine IPD surveillance or by review of patients' medical records.Another additional limitation of the PCV13 programme implemented in Cameroon is that it relies on an accelerated primary vaccination schedule with no booster.

Comment #2. The study population included children aged from 2 to 59 months during the period (August 2011 to December 2018) …Please, remove “(August 2011 to December 2018)” as it is already said 12 lines after.

Response # 2: We thank the Reviewer for drawing our attention here as well. This has been removed from the text.

Comment #3. If this is a retrospectively laboratory-based surveillance study, how were signed informed consents obtained from the parents/caretakers?

Response # 3:We thank the Reviewer for the chance to respond to this question. Yes, it was a retrospective, laboratory-based, surveillance study, and details are provided in the Methods section 2.1, which reads:Additionally, during the review of patients’ medical records and specific follow up of cases identified via routine IPD surveillance, vaccine history and signed informed consent forms were obtained from the parents/caretakers of each participating child.

As we also explained in the response to Major Comment #3, to retrieve comprehensive data (including vaccination history), we attempted to reach those parents/caretakers whose contact details were available in the registers. We reported what was found, being transparent in reporting exactly what proportion of children were identified with documented evidence of PCV13 vaccination through contact tracing, either by specific follow-up of vaccination status for cases that had been identified through routine IPD surveillance or by review of patients' medical records.

Comment # 4. The authors stated that “3-dose schedule (6, 10 and 14 weeks of age; 3+0) on date without any catch-up doses for older children” and later that “However, only children in the 2- to 11-month-old age bracket are eligible to receive free vaccination in the country.”
These two sentences are somehow contradictory; for instance, is a child of 6 months of age (24 weeks old) vaccinated or not?

Response # 4: We agree with the Reviewer that these two sentences might appear to be contradictory. Our intention here was to explain that, in the national immunization programme (NIP) in Cameroon, the age-group based approach differed between PCV13 and the traditional pediatric NIP vaccines. For the traditional pediatric NIP vaccines, children in the 2-to 11-month-old age group were eligible to receive free vaccination; whereas, for the PCV13 implementation programme,only those who were up to months age were eligible. Those older than 4 months were not vaccinated with PCV13. We observed this situation on the field were some children had their vaccination cards/booklets stamped with DTP1, DTP2 and DTP3 but were missing some doses of PCV13, although these were supposed to be administered simultaneously.

For clarification, we have complemented this statement with an additional sentence in Section 2.2, line 20 – 21 which now reads: ...although no catch-up vaccinations for PCV13 were planned for infants beyond 4months old.)

Comment # 5. Please, spell out Mother and Child Hospital (MCH) the first time it appears in the text.

Response # 5: This has been updated.

Comment # 6. Identification of S. pneumoniae from CSF by culture, …. Culture is not an identification technique. How were S. pneumoniae identified after cultured? Optochin test? Bili-solubility test?

Response # 6:We thank the Reviewer for drawing our attention to this, which has been revised in the text in section 2.5, lines 10-11with the following sentence:Colonies suspected to represent *S. pneumoniae* were confirmed using the optochin susceptibility test (5µg optochin disc; Oxoid) with respect to the manufacturer's instructions.

Comment # 7. Serotyping. The authors must include the serotyping real-time PCR used or at least a reference for the real-time PCR used for serotyping. How are serogroup 6 isolates identified? 6A/6B? 6A? What happens with serogroups 6C and 6D?

Response # 7: The text on serotyping has been revised (see section 2.6)

**Serotyping of pneumococcal isolates**

At the WHO/RRL, real-time polymerase chain reaction (PCR) detection technique was used for serotyping. As earlier reported (Kwambana-Adams et al., 2016), RNAse P gene assay was applied on all CSF specimens to confirm that the samples were of human origin and to monitor the

efficiency of amplification, which consisted of a series of cycles. First, there was an initial denaturation step at 95^0^C for 10 minutes, followed by 45 cycles of 95^0^C for 15 seconds and 60^0^C for 60 seconds. For each cycle of the targets, positivity was deduced using a cycle threshold (Ct) values, and Ct values of ≤36 were considered positive.

A CSF aliquot of 200 µl was added to 50 µl of TE buffer containing 0.08 g/ml of lysozyme (Sigma, L-6876) and 150 U/ml of mutanolysin (Sigma, M-9901), and the mixture was incubated for 1 hour at 37^0^C. Next, purified DNA extracts were exposed to a sequential triplex quantitative PCR (qPCR) assay designed to detect 21 pneumococcal capsular serotypes/serogroups using the African scheme as earlier reported (Pimenta et al., 2013). Pneumococci with Ct values ≤32 by qPCR that could not be serotyped/serogrouped under this African scheme were serotyped by conventional multiplex serotyping PCR assays. (Serotype data for other pathogens were not available.)

Comment # 8. “Purulent meningitis was defined as suspected meningitis with a CSF white blood
cell (WBC) count ≥20 per mm3.” However, in reference 18 of the WHO they make the following definition: Leukocytosis (> 100 cells/mm3); Leukocytosis (10-100 cells/ mm3) AND either an elevated protein (>100 mg/dl) or decreased glucose (< 40 mg/dl).

Response # 8: We agree with the Reviewer with the widely used WHO case-definition which we had referenced. However, as is common throughout West Africa, CSF glucose and protein measurements were not available.

To address the Reviewer's concern, we added the following text (see section 2.3 Case Definitions, lines 3 to 6):Purulent meningitis was defined as suspected meningitis with a CSF white blood cell (WBC) count ≥20/mm^3"^. Although WHO also includes elevated protein or decreased glucose as part of the case definition, these tests were not routinely performed in Cameroon, as is true in much of West Africa.

Comment # 9. What antigen (trademark) was used for pneumococcal detection? Could all antigen positive CSF be typed by real-time PCR?

Response # 9: The antigen used for pneumococcal detection was BinaxNOW^TM^. This has been updated in the text, see section 2.5, line 9.

We also wish to mention that the antigen tests used at the MCH or RRL (BinaxNOW^TM^ or PASTOREX^TM^) do allow for all antigen-positive CSF to be typed by real-time PCR.

Comment # 10. During the early years of SURVAC only culture or antigen positive specimens were sent…Please, define “early years” (2011 to 2014?)

Response # 10: We thank the Reviewer for drawing our attention to this. We have updated this, and the sentence now reads: “During the first years (2011 to 2012) of SURVAC only culture or antigen positive specimens were sent…” (see section 2.5, line 17.)

Comment#11.Analyses were conducted with SPSS version 25.0.Please, include the developing corporation of this software.

Response # 11: This has been updated and now reads "Analyses were conducted using the International Business Machines Corporation (IBM) Statistical Package for Social Sciences (IBM SPSSStatistics 25.0)".(See section 2.7)

Comment # 12. “(Fig 1. Profile of Purulent meningitis (WBC counts in CSF) in 2-59 months old children hospitalized in Yaoundé: 2011 - 2018. Note: [PCV13= 13-valent pneumococcal conjugate vaccine; CSF = Cerebrospinal fluid; WBC = White blood cell])”. I suggest deleting all the text after Fig. 1 and place it in the space corresponding to figure legends and include the “Note” as a footnote of the figure. What is the meaning of IBD (secondary vertical axe?) Please, write the figure of cases among hospitalized patients (secondary axe) with two figures the symbol %. Add to the legend what does the blue line mean.

Response # 12: We thank the Reviewer for the suggestions provided with regards to Fig 1. We had formatted Fig. 1 initially as the Reviewer now suggests, but the editorial staff recommended that we follow Journal recommendations. We would be ready to modify this Figure again if the editorial staff deem it necessary.

As requested, "the blue line" (which is now red colored in the updated figure) represents the percentage of purulent meningitis cases among all hospitalized patients. For clarity, we have revised the secondary vertical axis to align with the case definition, purulent meningitis. Therefore, the red line represents the percentage of cases (purulent meningitis) among all hospitalized patients. All other modifications have been made as suggested by the Reviewer. [See table at end of this Major Comments section.]

Comment #13. Tables 1a and 1b. There are a lot of cases with missing clinical information, at least more than 50%: 1629 diagnosis unknown, 1617 undocumented outcome at discharged, 2840 vaccine status unknown…How representative is the study herein presented?

Response # 13: We again agree with the Reviewer on this. Our analyses were basically descriptive because, as noted in our response to Major Comment #3, challenges with missing data are a phenomenon in most of resource-limited countries. To address the Reviewer's concern, we have added in the Discussion as a limitation that: “missing data may have impacted representativeness of our findings. However, since most children in the study, and in Cameroon, are living under the poverty line, their risk factors are likely broadly similar for disease outcomes and vaccination status".

Comment #14. To which dates correspond the sub-set of CSF specimens (N=765) subsequently tested using either microbiological culture, antigen detection and/or polymerase chain reaction (PCR)? EPP or LPP? It is impossible to know the representativeness of the data if just 765/3131 cases (less than 25%) were studied unless they were homogenously distributed through the years of the study. How many cases was form Yaounde and how many from outside (Table 4)? How many were studied each year (or at least each period) from within/outside Yaounde? It is very strange that there were 103 cases of S. pneumoniae meningitis and only 5 cases of N. meningitidis meningitis.

Response # 14: The CSF specimens sub-set corresponds to the study period from August 2011 to December 2018. Because of limited logistic supplies the whole sets of specimens could not be analyzed; therefore, the method used was to randomly select a quarter of samples from each year for analyses.

We have updated Table 2 with this information and explained elsewhere in the text that a representative subset of samples was chosen annually, for the years between 2012 and 2018. (By contrast, in the partial year2011, when PCV13 was introduced in July, all the available 47 CSF specimens were shipped to WHO/RRL.)

Comment # 15. “During the early years of SURVAC only culture or antigen positive specimens were sent to the WHO/RRL, but later all suspected isolates were shipped (Table 2).”
However in Table 2 in 2011 there are 3 PCR positive pneumococcal cases and only 1 by culture, and in 2013 there were 26 positives by PCR and only 13 by culture and 3 by antigen detection.

Response # 15: We agree with the Reviewer on the points been raised here. Specimens were sent as isolates (culture-positive) or CSF sample (antigen-positive).

The practice at the WHO/RRL is that all isolates/samples received are tested. Firststep was to confirm if the specimens originate from humans. Secondly, all culture-positive isolates initially from Cameroon were to be confirmed with PCR. Since all isolates and samples were retested at the WHO/RRL, we have reported the PCR confirmed outcome as the final diagnostic method for pneumococcal detection. (Additional text has been added in the manuscript for clarity).

Comment # 16. The distribution of cases in Table 2 seems somehow heterogeneous (range 47-662 cases). Have the authors any explanation for this situation? Please comment in the results or in the discussion.

Response # 16: PCV13 programme entered Cameroon at the start of July 2011. Our study period spans from August 2011 to December 2018. (2011 was a partial year becausePCV13was introduced in July.)

In the manuscript(Panel 2.), we have highlighted the lack of robustness in the surveillance programme in Cameroon to explain this heterogeneity. In particular, the surveillance programme performance was affected with factors/indicators such as:

1. Supply of reagents: Since the SURVAC project was not self-supported but relied entirely on stock supplies from partners, supplies were not usually timely and, in some cases, this delayed analyses.
2. Reporting and completion of entries: Because electronic data is not maintained, needing to track caregivers to get clinical/vaccine history was problematic. Hence, this contributed to the missing data scenario.
3. Consistency: The use of the international classification of diseases (ICD) codes is not mandatory in Cameroon, and this made it challenging to apply standard case definitions on clinical diagnosis.

Comment # 17. “The WHO/RRL in The Gambia did not use Quellung but rather PCR for serotyping, which in some cases did not distinguish individual serotypes (for example, 18A/18B/18C/18F).” However, in Table 3 serotype 18C is distinguished from other serogroup 18 serotypes. Also, serotype 6A/6B appear together but serotype 6A also appears alone.

Response # 17: We thank the Reviewer for this observation. As was earlier mentioned in Major Comment #2, this description that could include both serotypes and serogroups was a result of the method used. To recapitulate, purified DNA extracts were exposed to sequential triplex quantitative PCR (qPCR) assay designed to detect 21 pneumococcal capsular serotypes/serogroups using the African scheme as reported earlier(Pimenta et al., 2013).Pneumococci with Ct values ≤32 by qPCR that could not be serotyped/serogrouped under this African scheme were sequentially serotyped by conventional multiplex serotyping PCR assays. As the Reviewer notes, the WHO/RRL follows this Africa scheme approach.

| **Reaction no.** | **Africa** |
| --- | --- |
| 1 | 1, 5, 23F |
| 2 | 4, 6A/6B/6C/6D, 9V/9A |
| 3 | 14, 18C/18A/18B/18F, 19F |
| 4 | 3, 7F/7A, 19A |
| 5 | 6C/6D, 12F/12A/12B/44/46, 22F/22A |
| 6 | 15A/15F, 23A, 33F/33A/37 |
| 7 | 2, 11A/11D, 16F |

As noted in the Major Comment #2 Response, “pneumococci with Ct values ≤32 by qPCR that could not be serotyped/serogrouped under this African scheme were serotyped by conventional multiplex PCR assays”.Therefore, 18C and 6A appear alone.

As mentioned for Major Comment #3, with respect to the vaccine uptake, likewise for the WHO / RRL output, we “reported what was found, being transparent in reporting exactly” the serotyping results that were provided by the laboratory (We have added as a footnote in Table 3 that:The serotypes that appear alone were done by PCR since the African scheme did not consistently yield a serotype.)

Comment # 18. “Of the 103 children with pneumococcal meningitis evaluated for serotype, a vaccine serotype was present in 62% (31/50) of cases in the EPE versus 35.8% (19/53) in the LPE (Table 3).” Please, include the statistic p-value for this comparison.

Response # 18: We have included the statistic p-value for this comparison and the sentence now reads:As shown in Table3, of the 103 children with pneumococcal meningitis evaluated for serotype, a vaccine serotype was present in 62% (31/50) of cases in the EPE versus 35.8% (19/53) in the LPE, showing a statistically significant difference between the two periods (p = 0.0081).

The statistical analysis method applied is described in Section 2.6 of the manuscript.

Comment # 19. “Table 4. Comparison of some baseline/serotype characteristics between the 'early and late post-PCV13 impact…” I suggest re-titling the Table as “Table 4. Comparison of some epidemiological characteristics and serotype distribution between the 'early and late post-PCV13 impact…” And replace “PCV13 Serotype status” with “PCV13 serotypes”.

Response # 19: We thank the Reviewer for the suggestions. Table 4 now reads, "Comparison of some epidemiological characteristics between the 'early and late post-PCV13 impact' periods of 2-to 59-month-old children with laboratory-confirmed pneumococcal meningitis hospitalized at the surveillance hospitals in Yaoundé (N=103): 2011 -2018".

Comment # 20. “Serotype-specific distribution differed between the EPE and LPE, respectively; 6A/6B (24%), 5 (6%) and 6A (6%) versus 14 (13.2%), 3 (7.6%), 4 (5.6%) and 18C (5.6%) (Table 4).” I would say that this distribution is depicted in Table 3 (but do not forget to include a reference to Table 4). Besides, are these differences statistically significant? Again, it is very strange to see the distribution of serogroup 6 isolates: 6A/6B and 6A alone… Does this mean that 6A/6B isolates are in fact serotype 6B isolates?

Response # 20: We thank the Reviewer again for drawing our attention to this. We have added in Table 3 two columns providing percentage difference estimates (with 95% confidence intervals) and the corresponding p-values. Table 4 has been referenced in the text as well.

With respect to the serogroup/serotype presentations, we have reported the serotype data as obtained from the WHO/RRL. While we agree with the Reviewer on the particularity of the distribution of isolates for serogroup 6 (and other serogroups), it reflects the application of the Africa scheme method. In the case of serogroup 6, for instance, Reaction no. 2 detects 6A/6B/6C/6D, while Reaction no. 4 detects 6C/6D.

As mentioned for Major Comment #3, with respect to the vaccine uptake, likewise for the WHO/RRL output, we "reported what was found, being transparent in reporting exactly" the serotyping results that were provided by the laboratory. (We have added as a footnote in Table 3 that:"The serotypes that appear alone were done by PCR since the African scheme did not consistently yield a serotype".

Comment # 21. “Despite these gains, we did not see changes in the proportion of CSF samples with purulence in our study.” The presence of purulence can be due to multiple pathogens, not only to S. pneumoniae.

Response # 21: We agree with the Reviewer that purulence can be due to any of several different pathogens, including *S. pneumoniae*. We have revised the sentence and it now reads:“With respect to purulent meningitis, we did not see changes in the proportion of CSF samples with purulence in this study, although purulence can be due to multiple pathogens, not only S*. pneumoniae*.”

Comment # 22. “This may reflect trends in serotype-specific pneumococcal circulation related to confounding factors such as time since vaccination, varying population immunity, changes in antibiotic use patterns, or phenotypic shifts in organism invasiveness.” This sentence is quite speculative if not supported by an appropriate reference.

Response # 22: We agree again with the Reviewer on this and the statement has now been referenced (Oligbu et al., 2019; Rodrigo C et al., 2014)

Comment # 23. “However, as outlined in Table 2, because PCR was used for pathogen identification and serotyping, we used the total number of confirmed *S. pneumoniae* result as obtained by the PCR technique that has generally been used for molecular characterization [11,12,22].” The number of CSF sent and studied to the WHO/RRL each year must be clearly described, for instance in Table 2. It is not very understandable why purulent CSF negative for pneumococcal infection were not studied by PCR to detect N. meningitidis or H. influenzae and other pathogens in the WHO/RRL center.

Response # 23:We agree with the Reviewer on this as well. Clarity has been provided in Table 2 on the number of CSF sent to WHO/RRL each year.

Funding remained a substantial challenge, which explains as well why only PCR was used. The WHO/RRL and SURVAC was substantially supported with funds from the United States Centers for Disease Control and Prevention, and when this was streamlined in 2011/2012, (coinciding with the time of IPD addition into the SURVAC network in August 2011) it obviously affected their capacity too. (We have added some text in the Discussion section, Panel 2).

**Panel 2. Some of the observed indicators/factors influencing surveillance programme performance in Cameroon**

1. **Supply of reagents:** Since the SURVAC project was not self-supported but relied entirely on stock supplies from partners, supplies were not usually timely and, in some cases, this delayed analyses.
2. **Reporting and completion of entries:** Because electronic data is not maintained, needing to track caregivers to get clinical/vaccine history was problematic. Hence, this contributed to the missing data scenario.
3. **Consistency:** The use of the international classification of diseases (ICD) codes is not mandatory in Cameroon, and this made it challenging to apply standard case definitions on clinical diagnosis.

Comment # 24.” Furthermore, in our study pathogen identification was not done routinely in a systematic fashion, which limited our ability to account appropriately for the sensitivity of each diagnostic method.”If pathogen identification was not done routinely, how can authors be sure that the decrease in VST or the increase in some VST is not due to a bias in the number of pathogens identified in each time-period?

Response # 24: Another good question from the Reviewer.

Pneumococcal carriage is a precursor to invasive pneumococcal disease (including meningitis), and the changes seen in carriage are most likely to be seen in disease. We had conducted a previous study on carriage on infants in this population and obtained similar trends. (Njuma Libwea J, Gröndahl-Yli-Hannuksela K, Kobela M, Toropainen M, Nyholm O, Ndombo PK, et al. Prevalence of pneumococcal nasopharyngeal colonization and serotypes circulating in Cameroonian children after the 13-valent pneumococcal conjugate vaccine introduction. Int J Infect Dis. 2020;98: 113–120. doi: 10.1016/j.ijid.2020.06.048).Although this cannot completely rule out the possibility of bias in the number of pathogens identified in each time-period, a decrease in the proportion of VST pneumococcal meningitis accompanied with a simultaneous increase in the proportion of NVST neumococcal meningitis is consistent with thisdocumented change in prevalence of pneumococcal serotype carriage in the era of conjugate vaccines.

Comment # 25. “By contrast, in Cameroon during the early years of the surveillance programme, only sentinel surveillance was performed.” What does this sentence mean? What kind of surveillance was done in the late years?

Response # 25:We thank the Reviewer for this observation. Our intention here was to state that while some other countries that initially started with sentinel surveillance have evolved to nation-wide surveillance, the situation has not changed in Cameroon since its inception (i.e., the early years). This sentence has now been revised and reads:By contrast, only sentinel surveillance has been performed in Cameroon since its inception.
