# Supplementary material for: Impact of 13-valent pneumococcal conjugate vaccine on laboratory-confirmed pneumococcal meningitis and purulent meningitis among children ˂5 years in Cameroon, 2011–2018
Source: PLoS One. 2021 Apr 15;16(4):e0250010. doi: 10.1371/journal.pone.0250010 (PMC8049353; doi:10.1371/journal.pone.0250010)
Supplement: Attachment — Submitted filename: Response to Reviewers_PONE-D-20-24718R1_20 MAR21.docx [file pone.0250010.s005.docx]

PONE-D-20-24718R1
Impact of 13-valent pneumococcal conjugate vaccine on laboratory-confirmed pneumococcal meningitis and purulent meningitis among children ˂ 5 years in Cameroon, 2011 - 2018
PLOS ONE

Dear Dr.John Njuma Libwea, 

Thank you for submitting your manuscript to PLOS ONE. After careful consideration, we feel that it has merit but does not fully meet PLOS ONE’s publication criteria as it currently stands. Therefore, we invite you to submit a revised version of the manuscript that addresses the points raised during the review process.

Please submit your revised manuscript by May 03 2021 11:59PM. If you will need more time than this to complete your revisions, please reply to this message or contact the journal office at [plosone@plos.org](mailto:plosone@plos.org). When you're ready to submit your revision, log on to [https://www.editorialmanager.com/pone/](https://urldefense.proofpoint.com/v2/url?u=https-3A__www.editorialmanager.com_pone_&d=DwQFaQ&c=UE1eNsedaKncO0Yl_u8bfw&r=FLGIKPNY_xSYZqyTXUJQEH0v1CsK33K-64KyoFi5s0A&m=HgDvybLMzCrDPQckl8JNR42feJOxCusWg4Slw5z1CH4&s=71m4u_xgspOfxD2v7JErcJCW1dpnh7mL3QcfysJxMTU&e=) and select the 'Submissions Needing Revision' folder to locate your manuscript file.

Please include the following items when submitting your revised manuscript:

- A rebuttal letter that responds to each point raised by the academic editor and reviewer(s). You should upload this letter as a separate file labeled 'Response to Reviewers'.
- A marked-up copy of your manuscript that highlights changes made to the original version. You should upload this as a separate file labeled 'Revised Manuscript with Track Changes'.
- An unmarked version of your revised paper without tracked changes. You should upload this as a separate file labeled 'Manuscript'.

If you would like to make changes to your financial disclosure, please include your updated statement in your cover letter. Guidelines for resubmitting your figure files are available below the reviewer comments at the end of this letter.

If applicable, we recommend that you deposit your laboratory protocols in protocols.io to enhance the reproducibility of your results. Protocols.io assigns your protocol its own identifier (DOI) so that it can be cited independently in the future. For instructions see: [http://journals.plos.org/plosone/s/submission-guidelines#loc-laboratory-protocols](https://urldefense.proofpoint.com/v2/url?u=http-3A__journals.plos.org_plosone_s_submission-2Dguidelines-23loc-2Dlaboratory-2Dprotocols&d=DwMFaQ&c=UE1eNsedaKncO0Yl_u8bfw&r=FLGIKPNY_xSYZqyTXUJQEH0v1CsK33K-64KyoFi5s0A&m=HgDvybLMzCrDPQckl8JNR42feJOxCusWg4Slw5z1CH4&s=a43iTw9avlAcHiQGN1Jwawyt9zvLe0HH8_RkdumGQmo&e=)

We look forward to receiving your revised manuscript.

Kind regards,

Silvia Ricci

Academic Editor

PLOS ONE

**Review Comments to the Author.**

Please use the space provided to explain your answers to the questions above. You may also include additional comments for the author, including concerns about dual publication, research ethics, or publication ethics. (Please upload your review as an attachment if it exceeds 20,000 characters)

Reviewer #2: All questions raised by this reviewer have been properly answered and I actually appreciate the effort made by the authors in improving the text with the new comments introduced.

I only have 3 minor comments:

Response: Dear Reviewer, we thank you for the appreciations and recognize the minor comments you have raised in our data. Additionally, given the paucity of data on PCV impact from West Africa, with some of the highest pneumococcal disease burdens globally, we think our results are an important contribution to the literature.

**Minor comments**

Comment #1. Abstract. “…EPE were 6A/6B (24%), 5 (6%) and 6A (6%),…”
As not all serogroup 6 could be typed to the serotype level, I suggest (only for the abstract) to add 6A + 6A/B serotypes, what should make a 6A/6B (30%) and delete 6A (6%).
As both serotypes 6A/6B are included in the PCV13 and many molecular tests don’t differentiate between them, the reading of the abstract will be easier. In the text, with the explanations given, the results can be left as they are, differentiating between 6A and 6A/6B serotypes.

Response # 1: We thank the Reviewer for this suggestion, and this has now been revised in the Abstract to read; " The most frequent pneumococcal meningitis VSTs during the EPE were 6A/6B (30%) and 5 (6%), and during the LPE were 14 (13.2%), 3 (7.6%), 4 (5.6%) and 18C (5.6%)".

Comment #2. Page 6. “Pneumococci with Ct values ≤32 by qPCR that could not be serotyped/serogrouped under this African scheme were serotyped by conventional multiplex serotyping PCR assays.” Please, add a reference for the conventional multiplex serotyping PCR assays used.

Response # 2: The corresponding reference to this has now been added (See Section 2.6, line 14).

Comment # 3. Page 15. “We had considered this as a potential source of bias to our findings, but as documented with the prevalence of pneumococcal carriage in the era of conjugate vaccines, our data is consistent with the trends of decrease in some VST accompanied with a simultaneous increase in some NVST.” Please, provide a reference for the prevalence of pneumococcal carriage in the era of conjugate vaccines.

Response # 3: The references text have been added (See Discussion section, paragraph, line 51).
