# Supplementary material for: Impact of 13-valent pneumococcal conjugate vaccine on laboratory-confirmed pneumococcal meningitis and purulent meningitis among children ˂5 years in Cameroon, 2011–2018
Source: PLoS One. 2021 Apr 15;16(4):e0250010. doi: 10.1371/journal.pone.0250010 (PMC8049353; doi:10.1371/journal.pone.0250010)
Supplement: Peer review history

# Peer review: Decision Letter 0

**DOI:** 10.1371/journal.pone.0250010.r001

**Authors:** Silvia Ricci

17 Nov 2020

PONE-D-20-24718

Impact of 13-valent pneumococcal conjugate vaccine in Cameroon on laboratory-confirmed pneumococcal meningitis and purulent meningitis  among children ˂ 5 years,  2011 - 2018

PLOS ONE

Dear Dr. John Njuma Libwea

Thank you for submitting your manuscript to PLOS ONE. After careful consideration, we feel that it has merit but does not fully meet PLOS ONE’s publication criteria as it currently stands. Therefore, we invite you to submit a revised version of the manuscript that addresses the points raised during the review process.

Please submit your revised manuscript by 09.12.20. If you will need more time than this to complete your revisions, please reply to this message or contact the journal office at plosone@plos.org. When you're ready to submit your revision, log on to https://www.editorialmanager.com/pone/ and select the 'Submissions Needing Revision' folder to locate your manuscript file.

Please include the following items when submitting your revised manuscript:

- A rebuttal letter that responds to each point raised by the academic editor and reviewer(s). You should upload this letter as a separate file labeled 'Response to Reviewers'.
- A marked-up copy of your manuscript that highlights changes made to the original version. You should upload this as a separate file labeled 'Revised Manuscript with Track Changes'.
- An unmarked version of your revised paper without tracked changes. You should upload this as a separate file labeled 'Manuscript'.

If you would like to make changes to your financial disclosure, please include your updated statement in your cover letter. Guidelines for resubmitting your figure files are available below the reviewer comments at the end of this letter.

If applicable, we recommend that you deposit your laboratory protocols in protocols.io to enhance the reproducibility of your results. Protocols.io assigns your protocol its own identifier (DOI) so that it can be cited independently in the future. For instructions see: http://journals.plos.org/plosone/s/submission-guidelines#loc-laboratory-protocols

We look forward to receiving your revised manuscript.

Kind regards,

Silvia Ricci

Academic Editor

PLOS ONE

Journal Requirements:

When submitting your revision, we need you to address these additional requirements.

1. Please ensure that your manuscript meets PLOS ONE's style requirements, including those for file naming. The PLOS ONE style templates can be found at

https://journals.plos.org/plosone/s/file?id=wjVg/PLOSOne_formatting_sample_main_body.pdf and

https://journals.plos.org/plosone/s/file?id=ba62/PLOSOne_formatting_sample_title_authors_affiliations.pdf

2. Thank you for stating the following in the Competing Interests section: 

"I have read the journal's policy and the authors of this manuscript have the following competing interests: [Some of the co-authors are employees of Pfizer but their independent contributions do not represent those of their institution]".

i) Please confirm that this does not alter your adherence to all PLOS ONE policies on sharing data and materials, by including the following statement: "This does not alter our adherence to  PLOS ONE policies on sharing data and materials.” (as detailed online in our guide for authors http://journals.plos.org/plosone/s/competing-interests).  If there are restrictions on sharing of data and/or materials, please state these. Please note that we cannot proceed with consideration of your article until this information has been declared.

ii) Please include your updated Competing Interests statement in your cover letter; we will change the online submission form on your behalf.

Please know it is PLOS ONE policy for corresponding authors to declare, on behalf of all authors, all potential competing interests for the purposes of transparency. PLOS defines a competing interest as anything that interferes with, or could reasonably be perceived as interfering with, the full and objective presentation, peer review, editorial decision-making, or publication of research or non-research articles submitted to one of the journals. Competing interests can be financial or non-financial, professional, or personal. Competing interests can arise in relationship to an organization or another person. Please follow this link to our website for more details on competing interests: http://journals.plos.org/plosone/s/competing-interests

3. Please amend either the title on the online submission form (via Edit Submission) or the title in the manuscript so that they are identical.

4. Please include captions for your Supporting Information files at the end of your manuscript, and update any in-text citations to match accordingly. Please see our Supporting Information guidelines for more information: http://journals.plos.org/plosone/s/supporting-information. 

5. Please include your tables as part of your main manuscript and remove the individual files. Please note that supplementary tables (should remain/ be uploaded) as separate "supporting information" files

**Comments to the Author**

1. Is the manuscript technically sound, and do the data support the conclusions?

The manuscript must describe a technically sound piece of scientific research with data that supports the conclusions. Experiments must have been conducted rigorously, with appropriate controls, replication, and sample sizes. The conclusions must be drawn appropriately based on the data presented. 

Reviewer #1: Partly

**********

2. Has the statistical analysis been performed appropriately and rigorously? 

Reviewer #1: No

**********

3. Have the authors made all data underlying the findings in their manuscript fully available?

The PLOS Data policy requires authors to make all data underlying the findings described in their manuscript fully available without restriction, with rare exception (please refer to the Data Availability Statement in the manuscript PDF file). The data should be provided as part of the manuscript or its supporting information, or deposited to a public repository. For example, in addition to summary statistics, the data points behind means, medians and variance measures should be available. If there are restrictions on publicly sharing data—e.g. participant privacy or use of data from a third party—those must be specified.

Reviewer #1: No

**********

4. Is the manuscript presented in an intelligible fashion and written in standard English?

PLOS ONE does not copyedit accepted manuscripts, so the language in submitted articles must be clear, correct, and unambiguous. Any typographical or grammatical errors should be corrected at revision, so please note any specific errors here.

Reviewer #1: Yes

**********

5. Review Comments to the Author

Please use the space provided to explain your answers to the questions above. You may also include additional comments for the author, including concerns about dual publication, research ethics, or publication ethics. (Please upload your review as an attachment if it exceeds 20,000 characters)

Reviewer #1: Impact of 13-valent pneumococcal conjugate vaccine in Cameroon on laboratory-confirmed pneumococcal meningitis and purulent meningitis among children < 5 years, 2011-2018

The authors conducted a retrospective observational study in patients aged< 5 yrs hospitalized for clinically suspected meningitis (CSBM,) admitted to hospitals in Yaoundè, capitol city of Cameroon, from August 2011 to December 2018. They declared aim of this study was to describe the impact of PCV13 on serotype distribution among laboratory confirmed pneumococcal meningitis cases over this period of time.

General comment

The authors report well known published information on pneumococcal meningitis from neighboring countries of the African meningitis belt, before and after the introduction of PCV-13. However, their article presents many shortcomings in data collection, description of laboratory testing, statistical analyses and results.

Major comments

• The study evaluated children born before the introduction of PCV-13 and does not specify which children had been vaccinated with PCV-13 and which one had received PCV-7.

Authors declare that in Cameroon, since 2012, uptake for the third dose of PCV-13has been over 80%. However, in their study it is not emphasized that only 9.7% of children had a vaccination history. This approach offers a fundamental bias for evaluating objectively the effect of a correct immunization program on the epidemiology of meningitis.

• The authors mentioned that microbiological identification was obtained through bacterial culture, antigen testing and PCR. Unfortunately, incomplete information on the number of test performed and their sensitivity was not reported.

Methods

• Please provide some information regarding the study site (size of hospitals, type of hospitals (primary/secondary/tertiary care, pediatric care, for what population they are a referral center, etc..)

• The authors assert to have evaluated the “epidemiology” of pneumococcal meningitis during periods of PCV13 implementation in Cameroon. However, data are available only for 103 patients.

Did the authors calculate the power of the study and a sample size? The missing data may affect the generalizability of results.

Results

• The data about purulent meningitis is not clear. The authors defined purulent meningitis as “suspected meningitis with a CSF white blood cell (WBC) count � 20 per mm3”. In the results the percentage of EEP and LPP is calculated on a total of 3131 purulent meningitis (1486 + 1645) but in Table 1b only 266 cases meet the criteria. Can the authors explain better the eligibility criteria and the selection of study participants?

• It would be better, if possible, that authors provide characteristics of the 103 study participants (e.g., demographic, age, …)

• It is appropriate to summarize asymmetrical distribution (as data in table 1a, table 1 b, table 3, table 4) by giving the median and percentile range (for example, 25th and 75th percentiles, giving the mean and standard deviation for continuous variable and to indicate the missing data for each variable.

• Table 1 b: the title describes the “characteristics aged 2-59 months evaluated for suspected meningitis”. In the table the number of suspected meningitis is 3026.

• Table 3: the title quotes a total of 105 participants (N = 105), actual total of results is 103.

• Reference 13 is not appropriate at the point of the discussion.

Discussion

• It is recommended to discuss in more detail the limitations of the study, taking into account sources of potential bias or shortcomings (study size, confounders, etc) and provide a conservative overall interpretation of data.

• Authors may compare the study being presented with other studies in the literature in terms of validity, generalizability, and precision.

Despite the laudable effort of the AA in collecting data, the study has not the power to add significant evidence on Pneumococcal meningitis trends in the contest of PCV-13 and to support a change in the vaccination schedule and in the understanding of Pneumococcal Meningitis epidemiology in Cameroon due the above expressed limits.

**********

6. PLOS authors have the option to publish the peer review history of their article (what does this mean?). If published, this will include your full peer review and any attached files.

If you choose “no”, your identity will remain anonymous but your review may still be made public.

**Do you want your identity to be public for this peer review?** For information about this choice, including consent withdrawal, please see our Privacy Policy.

Reviewer #1: No

[NOTE: If reviewer comments were submitted as an attachment file, they will be attached to this email and accessible via the submission site. Please log into your account, locate the manuscript record, and check for the action link "View Attachments". If this link does not appear, there are no attachment files.]

While revising your submission, please upload your figure files to the Preflight Analysis and Conversion Engine (PACE) digital diagnostic tool, https://pacev2.apexcovantage.com/. PACE helps ensure that figures meet PLOS requirements. To use PACE, you must first register as a user. Registration is free. Then, login and navigate to the UPLOAD tab, where you will find detailed instructions on how to use the tool. If you encounter any issues or have any questions when using PACE, please email PLOS at figures@plos.org. Please note that Supporting Information files do not need this step.

Attachment Submitted filename: Revisione .docx Click here for additional data file.

# Author comment: Author response to Decision Letter 0

**DOI:** 10.1371/journal.pone.0250010.r002

8 Dec 2020

Impact of 13-valent pneumococcal conjugate vaccine in Cameroon on laboratory-confirmed pneumococcal meningitis and purulent meningitis among children < 5 years, 2011-2018

The authors conducted a retrospective observational study in patients aged <5yrs hospitalized for clinically suspected meningitis (CSBM,) admitted to hospitals in Yaoundé, capitol city of Cameroon, from August 2011 to December 2018. They declared aim of this study was to describe the impact of PCV13 on serotype distribution among laboratory confirmed pneumococcal meningitis cases over this period. 

General comment

The authors report well known published information on pneumococcal meningitis from neighbouring countries of the African meningitis belt, before and after the introduction of PCV-13. However, their article presents many shortcomings in data collection, description of laboratory testing, statistical analyses, and results.

_____________________________________________________________________________

Dear Reviewer, we recognize the limitations in our data, but nevertheless think that they are robust for the analysed outcomes. Additionally, given the paucity of data on PCV impact from West Africa, with some of the highest pneumococcal disease burdens globally, we think our results are an important contribution to the literature.

Major comments

Comment No. 1:The study evaluated children born before the introduction of PCV-13 and does not specify which children had been vaccinated with PCV-13 and which one had received PCV-7.

Response No. 1:The highlighted following text has now been included in the 4th paragraph, lines 8 to 10 in the background section to provide clarity: "Prior to PCV13 introduction in July 2011 in Cameroon, no pneumococcal conjugate vaccination (neither PCV7 nor PCV10) was included in the national immunization programme". In addition, in this study we evaluated only children born from August 2011 onwards who were, therefore, all eligible for PCV13 vaccination. 

Comment No. 2:Authors declare that in Cameroon, since 2012, uptake for the third dose of PCV-13 has been over 80%.However, in their study it is not emphasized that only 9.7% of children had a vaccination history. This approach offers a fundamental bias for evaluating objectively the effect of a correct immunization program on the epidemiology of meningitis.

Response No. 2: We thank the reviewer for this observation. The following statement on this has now been included (Results section, 1st paragraph line 4): “…and 9.3% of the total number enrolled had a documented PCV13 vaccination history”. 

We have also added additional text in the Discussion (page 15, see 4th paragraph, lines 3 to 8): "The lack of robust vaccination history means that fewer than 80% of children may have been vaccinated, potentially explaining why more dramatic reductions weren't seen.It may also suggest limitations of the PCV13 programme as implemented in Cameroon, either due to accelerated primary vaccination schedule with no booster or possibly because of vaccine uptake levels that were less than those reported by the administration. The latter would explain the frequent identification of PCV13 VSTs during the LPE.”

Comment No. 3:The authors mentioned that microbiological identification was obtained through bacterial culture, antigen testing and PCR. Unfortunately, incomplete information on the number of tests performed and their sensitivity was not reported.

Response No. 3: We have provided additional information on why this was so. The following sentence has been included in section 2.5, lines 7-8: "During the early years of SURVAC, only culture or antigen positive specimens were sent to the WHO/RRL but later all suspected specimens were shipped (Table 2)". Also, we have updated the results section 3.2 with corresponding information to support the ensuing statement from section 2.5: Only a sub-set of CSF specimens (N=765), were subsequently found to have been tested using either microbiological culture, antigen detection and/or polymerase chain reaction (PCR). Of this subset of 765 CSF specimens, 142 were laboratory confirmed as cases of bacterial meningitis including 103 resulting from S. pneumoniae, 13 from H. influenzae, 5 of N. meningitidis and 21 were a collection of other organisms.

Methods

Comment No. 4:Please provide some information regarding the study site (size of hospitals, type of hospitals (primary/secondary/tertiary care, pediatric care, for what population they are a referral center, etc..).

Response No. 4:

The following description has been added to the manuscript (see section “2.2 Study design, setting and selection of study participants”, paragraph 1, lines 4 to 13): "As previously described [5], study sites hosted at the paediatric reference hospital in Yaounde (MCH) constitute a group of primary and secondary healthcare institutions (covering localities situated within 80 km radius from Yaoundé, Cameroon's capital city) that were involved with the sentinel surveillance of invasive diseases. Yaoundé and its surroundings have a population of over 3.5 million, of which 18% were children aged under-five years based on 2010 National Population Census. The MCH is one of the largest children’s hospitals in the country and it is accessible and affordable to the local population. MCH keeps records of hospital visits, admissions, and deaths, in addition to specific clinical, laboratory and serotype data on invasive diseases including Haemophilus influenzae and Streptococcus pneumoniae. It has a capacity of 300 beds and records over 12000 admissions annually".

Comment No. 5:The authors assert to have evaluated the “epidemiology” of pneumococcal meningitis during periods of PCV13 implementation in Cameroon. However, data are available only for 103 patients.

Response No. 5:

We thank the reviewer again for this observation and acknowledge that the data available only for 103 children with a pneumococcal serotype may be sparse, but this could be explained when considering the surveillance programme limitations that are presented in the manuscript discussion. Also, we explained in section 2.5, lines 7 to 9 that, during the early years of SURVAC only culture or antigen positive specimens were sent to the WHO/RRL but later all suspected specimens were shipped. The text has been updated, so that the term epidemiology is removed from the first line of the discussion: "We evaluated changes in the distribution of serotypes causing penumococcal meningitis during the early and later periods of PCV13 implementation in Cameroon".( see Discussion section, paragraph 1, lines 1 to 2)

Comment No. 6:Did the authors calculate the power of the study and a sample size? The missing data may affect the generalizability of results. 

Response No. 6: Yes, we had estimations for sample size and power of the study in the statistical analysis plan because the initial goal was to conduct a study with both retrospective and prospective arms. But due to logistical challenges, the prospective arm was not feasible at the time. Therefore, since this study involved just the retrospective arm that used register-based laboratory data, the entire retrospective database was used and as this was a descriptive study, it made sample size calculations unnecessary. 

Results

Comment No. 7:The data about purulent meningitis is not clear. The authors defined purulent meningitis as “suspected meningitis with a CSF white blood cell (WBC) count � 20 per mm3”. In the results the percentage of EEP and LPP is calculated on a total of 3131 purulent meningitis (1486 + 1645) but in Table 1b only 266 cases meet the criteria. Can the authors explain better the eligibility criteria and the selection of study participants? 

Response No. 7: We thank the reviewer again for drawing our attention to this. 

The study population included children aged from 2 to 59 months during the period (August 2011 to December 2018) who were admitted at any of the five surveillance hospitals within the Surveillance Epidemiologique en l'Afrique Central (SURVAC) network in Yaounde, and who had cerebrospinal fluid (CSF) collected for suspected bacterial meningitis (see section 2.2, second paragraph, lines 14 to 18).

Moreover, subject selection was based on any child meeting the inclusion criteria with a lumbar puncture performed and a CSF WBC result available.

The correct definition of purulent meningitis was included, “suspected meningitis with a CSF white blood cell (WBC) count � 20 per mm3”. However, we had omitted to define in the text what was not considered as a purulent meningitis case (i.e. non-cases). This has now been added to the inclusion criteria text in the manuscript, section "2.3 Case definition": "Patients with a CSF WBC count <20 per mm3 were not considered to have purulent meningitis" (see section 2.3, paragraph 1 and line 4).

Comment No. 8:It would be better, if possible, that authors provide characteristics of the 103 study participants (e.g., demographic, age, …)

Response No. 8: This is provided for gender, age group, and place of residence (See Table 4).

If the Editors or Reviewer have additional items they would like to include, please let us know, although we may have limited ability to access these data.

Comment No. 9:It is appropriate to summarize asymmetrical distribution (as data in table 1a, table 1 b, table 3, table 4) by giving the median and percentile range (for example, 25th and 75th percentiles, giving the mean and standard deviation for continuous variable and to indicate the missing data for each variable.

Response No. 9: We agree with the Reviewer on the appropriateness of presenting asymmetric data by giving median and percentile range, but with mean and standard deviation for continuous variables (Categorizing the continuous variables {e.g. age, WBC counts}, as we have done, was to facilitate comparison with previous studies).

This has been updated were appropriate in the tables specified by the Reviewer. With respect to the missing data, this has been documented in Table 1a for "PCV13 vaccination status" ("Undocumented") and Table 1b for "Diagnosis at time discharge" ("Unknown {missing data}"), "Previous antibiotic use prior to admission" ("Unknown"), "CSF appearance" ("Others") and "Outcome at discharge" ("Undocumented").

Comment No. 10:Table 1 b: the title describes the “characteristics aged 2-59 months evaluated for suspected meningitis”. In the table the number of suspected meningitis is 3026.

Response No. 10: We thank the Reviewer for drawing our attention to this. Our intention was to describe the situation at the time of patient discharge from the hospital. This has now been modified in the manuscript, and the title reads "Clinical and laboratory outcomes of children aged 2 – 59 months evaluated for suspected meningitis admitted at the sentinel surveillance hospitals in Yaoundé: 2011 ─ 2018 (N = 3131)", and the category "Suspected meningitis" has been corrected to read "Diagnoses at time of discharge" 

We also thank the Reviewer for pointing out the difference in the number of suspected meningitis cases. There were 3026 cases identified at the time of triage, as compared to 3131 cases with a discharge diagnosis, which was the outcome used for this study.

Comment No. 11:Table 3: the title quotes a totalof 105 participants (N = 105), actual total of results is 103.

Response No. 11: There was a typographical error that has now been rectified. The total number of participants now reads, N=103

Comment No. 12:Reference 13 is not appropriate at the point of the discussion.

Response No. 12: The reference has been removed, and the reference has been renumbered. 

Discussion

Comment No. 13:It is recommended to discuss in more detail the limitations of the study,considering sources of potential bias or shortcomings (study size, confounders, etc) and provide a conservative overall interpretation of data. Authors may compare the study being presented with other studies in the literature in terms of validity, generalizability, and precision.

Response No. 13: This has been revised across the section. For instance, in the second paragraph under discussion, lines 1 -6; the sentence now reads "Despite these gains, we did not see changes in the proportion of CSF samples with purulence in our study. While this may reflect any substantial fluctuations in surveillance that occurred over the surveillance period, it could also suggest limitations of the PCV13 programme as implemented in Cameroon, either due to the accelerated schedule with no booster or possibly because of coverage levels that were less than those reported by the Ministry of Health. The latter may explain the frequent identification of PCV13 VSTs during the LPE."

There are additional discussion on the limitations on the third paragraph from line 4 to 16, as follows: "Sentinel surveillance was only initiated at the time of vaccine introduction, so data were not available from the pre-vaccination era, though this was undertaken in other Sub-Saharan countries [11,12,22]. Furthermore, in our study, pathogen identification was not done routinely in a systematic fashion, which limited our ability to account appropriately for the sensitivity of each diagnostic method. However, as outlined in Table 2, because PCR was used for pathogen identification and serotyping, we used the total number of confirmed S. pneumoniae result as obtained by the PCR technique that has generally been used for molecular characterization[11,12,22]. Surveillance was conducted only in the Yaounde area, potentially limiting generalizability of the findings. Burkina Faso for example, performs nation-wide meningitis surveillance and routine serotyping of all pneumococcal meningitis specimens. By contrast, in Cameroon during the early years of the surveillance programme, only sentinel surveillance was performed. Specimens were not serotyped locally and only culture-positive pneumococcal specimens were shipped to the WHO regional reference laboratory at the MRC in The Gambia for serotyping which may have resulted in a low proportion of pneumococcal serotypes being identified".

Attachment Submitted filename: Response to Reviewers_PLoS One _ 4 Dec 2020_final.docx Click here for additional data file.

# Peer review: Decision Letter 1

**DOI:** 10.1371/journal.pone.0250010.r003

**Authors:** Silvia Ricci

21 Jan 2021

PONE-D-20-24718R1

Impact of 13-valent pneumococcal conjugate vaccine on laboratory-confirmed pneumococcal meningitis and purulent meningitis among children ˂ 5 years in Cameroon,  2011 - 2018

PLOS ONE

Dear Dr.John Njuma Libwea, 

Thank you for submitting your manuscript to PLOS ONE. After careful consideration, we feel that it has merit but does not fully meet PLOS ONE’s publication criteria as it currently stands. Therefore, we invite you to submit a revised version of the manuscript that addresses the points raised during the review process.

Please submit your revised manuscript by Mar 07 2021 11:59PM. If you will need more time than this to complete your revisions, please reply to this message or contact the journal office at plosone@plos.org. When you're ready to submit your revision, log on to https://www.editorialmanager.com/pone/ and select the 'Submissions Needing Revision' folder to locate your manuscript file.

Please include the following items when submitting your revised manuscript:

- A rebuttal letter that responds to each point raised by the academic editor and reviewer(s). You should upload this letter as a separate file labeled 'Response to Reviewers'.
- A marked-up copy of your manuscript that highlights changes made to the original version. You should upload this as a separate file labeled 'Revised Manuscript with Track Changes'.
- An unmarked version of your revised paper without tracked changes. You should upload this as a separate file labeled 'Manuscript'.

If you would like to make changes to your financial disclosure, please include your updated statement in your cover letter. Guidelines for resubmitting your figure files are available below the reviewer comments at the end of this letter.

If applicable, we recommend that you deposit your laboratory protocols in protocols.io to enhance the reproducibility of your results. Protocols.io assigns your protocol its own identifier (DOI) so that it can be cited independently in the future. For instructions see: http://journals.plos.org/plosone/s/submission-guidelines#loc-laboratory-protocols

We look forward to receiving your revised manuscript.

Kind regards,

Silvia Ricci

Academic Editor

PLOS ONE

[Note: HTML markup is below. Please do not edit.]

Reviewers' comments:

Reviewer's Responses to Questions

**Comments to the Author**

1. If the authors have adequately addressed your comments raised in a previous round of review and you feel that this manuscript is now acceptable for publication, you may indicate that here to bypass the “Comments to the Author” section, enter your conflict of interest statement in the “Confidential to Editor” section, and submit your "Accept" recommendation.

Reviewer #2: (No Response)

**********

2. Is the manuscript technically sound, and do the data support the conclusions?

The manuscript must describe a technically sound piece of scientific research with data that supports the conclusions. Experiments must have been conducted rigorously, with appropriate controls, replication, and sample sizes. The conclusions must be drawn appropriately based on the data presented. 

Reviewer #2: Partly

**********

3. Has the statistical analysis been performed appropriately and rigorously? 

Reviewer #2: I Don't Know

**********

4. Have the authors made all data underlying the findings in their manuscript fully available?

The PLOS Data policy requires authors to make all data underlying the findings described in their manuscript fully available without restriction, with rare exception (please refer to the Data Availability Statement in the manuscript PDF file). The data should be provided as part of the manuscript or its supporting information, or deposited to a public repository. For example, in addition to summary statistics, the data points behind means, medians and variance measures should be available. If there are restrictions on publicly sharing data—e.g. participant privacy or use of data from a third party—those must be specified.

Reviewer #2: No

**********

5. Is the manuscript presented in an intelligible fashion and written in standard English?

PLOS ONE does not copyedit accepted manuscripts, so the language in submitted articles must be clear, correct, and unambiguous. Any typographical or grammatical errors should be corrected at revision, so please note any specific errors here.

Reviewer #2: Yes

**********

6. Review Comments to the Author

Please use the space provided to explain your answers to the questions above. You may also include additional comments for the author, including concerns about dual publication, research ethics, or publication ethics. (Please upload your review as an attachment if it exceeds 20,000 characters)

Reviewer #2: There are three main issues that must be clarified by the authors:

1. The samples studied and their distribution by year must be clear. It is impossible to know how many CSF samples of those obtained were studied each year, by what technique and their results. How many samples were from Yaounde or from outside each year of the study. This must be clearly depicted in Table 2.

2. Microbiological methods must be clearly described: what media were used for culture; what identification techniques were used, what antigen, what real-time PCR…

3. How is it possible that “since 2012, uptake for the third dose of DTwP/PCV13 in Cameroon has been over 80%[16]” and in the study only 9.3% of the total number enrolled had a documented PCV13 vaccination history (5.5% with 3 doses)?

Without clearly describing these issues it is impossible to know the representativeness of the sample and consequently, the value of the results obtained.

Other comments:

1. “We retrospectively analyzed laboratory-based surveillance data from August 2011 to December 2018.”

However Fig 1. represents data from 2011 to 2019. What happened in 2019? There were no cases of pneumococcal meningitis?

It would be desirable to have the incidence data of pneumococcal meningitis of both periods studied, to see if the introduction of vaccine has represented a decrease in the incidence of meningitis or not.

2.The study population included children aged from 2 to 59 months during the period (August 2011 to December 2018)…

Please, remove “(August 2011 to December 2018)” as it is already said 12 lines after.

3. If this is a retrospectively laboratory-based surveillance study, how were signed informed consents obtained from the parents/caretakers?

4. The authors stated that “3-dose schedule (6, 10 and 14 weeks of age; 3+0) without any catch-up doses for older children” and later that “However, only children in the 2 to 11month old age bracket are eligible to receive free vaccination in the country.”

These two sentences are somehow contradictory; for instance, is a child of 6 months of age (24 weeks old) vaccinated or not?

5. Please, spell out Mother and Child Hospital (MCH) the first time it appears in the text

6. Identification of S. pneumoniae from CSF either by culture, …. Culture is not an identification technique. How were S. pneumoniae identified after cultured? Optochin test? Bili-solubility test?

7. Serotyping. The authors must include the serotyping real-time PCR used or at least a reference for the real-time PCR used for serotyping. How are serogroup 6 isolates identified? 6A/6B? 6A? What happens with serogroups 6C and 6D?

8. “Purulent meningitis was defined as suspected meningitis with a CSF white blood

cell (WBC) count ≥20 per mm3.”

However, in reference 18 of the WHO they make the following definition:

Leukocytosis (> 100 cells/mm3);

Leukocytosis (10-100 cells/ mm3) AND either an elevated protein (>100 mg/dl) or decreased glucose (< 40 mg/dl).

9. What antigen (trade mark) was used for pneumococcal detection? Could all antigen positive CSF be typed by real-time PCR?

10. During the early years of SURVAC only culture or antigen positive specimens were sent…

Please, define “early years” (2011 to 2014?)

11. Analyses were conducted with SPSS version 25.0.

Please, include the developing corporation of this sofware.

12. “(Fig 1. Profile of Purulent meningitis (WBC counts in CSF) in 2-59 months old children hospitalized in Yaounde: 2011 - 2018. Note: [PCV13= 13-valent pneumococcal conjugate vaccine; CSF = Cerebrospinalfluid; WBC = White blood cell])”

I suggest to delete all the text after Fig. 1 and place it in the space corresponding to figure legends and include the “Note” as a footnote of the figure.

What is the meaning of IBD (secondary vertical axe?) Please, write the figure of cases among hospitalized patients (secondary axe) with two figures an the symbol %. Add to the legend what does the blue line mean.

13. Tables 1a and 1b. There are a lot of cases with missing clinical information, at least more than 50%: 1629 diagnosis unknown, 1617 undocumented outcome at discharged, 2840 vaccine status unknown…

How representative is the study herein presented?

14. To which dates correspond the sub-set of CSF specimens (N=765) subsequently tested using either microbiological culture, antigen detection and/or polymerase chain reaction (PCR)? EPP or LPP? It is impossible to know the representativeness of the data if just 765/3131 cases (less than 25%) were studied unless they were homogenously distributed through the years of the study.

How many cases was form Yaounde and how many from outside (Table 4)? How many were studied each year (or at least each period) from within/outside Yaounde?

It is very strange that there were 103 cases of S. pneumoniae meningitis and only 5 cases of N. meningitidis meningitis.

15. “During the early years of SURVAC only culture or antigen positive specimens were sent to the WHO/RRL, but later all suspected isolates were shipped (Table 2).”

However in Table 2 in 20111 there are 3 PCR positive pneumococcal cases and only 1 by culture, and in 2013 there were 26 positive by PCR and only 13 by culture and 3 by antigen detection.

16. The distribution of cases in Table 2 seems somehow heterogeneous (range 47-662 cases). Have the authors any explanation for this situation? Please comment in the results or in the discussion.

17. “The WHO/RRL in The Gambia did not use Quellung but rather PCR for serotyping, which in some cases did not distinguish individual serotypes (for example, 18A/18B/18C/18F).”

However, in Table 3 serotype 18C is distinguished from other serogroup 18 serotypes.

Also, serotype 6A/6B appear together but serotype 6A also appears alone.

18. “Of the 103 children with pneumococcal meningitis evaluated for serotype, a vaccine serotype was present in 62% (31/50) of cases in the EPE versus 35.8% (19/53) in the LPE (Table 3).”

Please, include the statistic p-value for this comparison.

19. “Table 4. Comparison of some baseline/serotype characteristics between the 'early and late post-PCV13 impact…”

I suggest retitling the Table as “Table 4. Comparison of some epidemiological characteristics and serotype distribution between the 'early and late post-PCV13 impact…”

And replace “PCV13 Serotype status” with “PCV13 serotypes”

20. “Serotypespecific distribution differed between the EPE and LPE, respectively; 6A/6B (24%), 5 (6%) and 6A (6%) versus 14 (13.2%), 3 (7.6%), 4 (5.6%) and 18C (5.6%) (Table 4).”

I would said that this distribution is depicted in Table 3 (but don’t forget to include a reference to Table 4). Besides, are these differences statistical significant? Again it is very strange to see the distribution of serogroup 6 isolates: 6A/6B and 6A alone… Does this mean that 6A/6B isolates are in fact serotype 6B isolates?

21. “Despite these gains, we did not see changes in the proportion of CSF samples with purulence in our study.”

The presence of purulence can be due to multiple pathogens, not only to S. pneumoniae.

22. “This may reflect trends in serotype-specific pneumococcal circulation related to confounding factors such as time since vaccination, varying population immunity, changes in antibiotic use patterns, or phenotypic shifts in organism invasiveness.”

This sentence is quite speculative if not supported by an appropriate reference.

23. “However, as outlined in Table 2, because PCR was used for pathogen identification and serotyping, we used the total number of confirmed S. pneumoniae result as obtained by the PCR technique that has generally been used for molecular characterization [11,12,22].”

The number of CSF sent and studied to the WHO/RRL each year must be clearly described, for instance in Table 2. It is not very understandable why purulent CSF negative for pneumococcal infection were not studied by PCR to detect N. meningitidis or H. influenzae and other pathogens in the WHO/RRL center.

24.” Furthermore, in our study pathogen identification was not done routinely in a systematic fashion, which limited our ability to account appropriately for the sensitivity of each diagnostic method.”

If pathogen identification was not done routinely, how can authors be sure that the decrease in VST or the increase in some VST is not due to a bias in the number of pathogen identified in each time-period?

25 “By contrast, in Cameroon during the early years of the surveillance programme, only sentinel surveillance was performed.”

What does this sentence mean? What kind of surveillance was done in the late years?

**********

7. PLOS authors have the option to publish the peer review history of their article (what does this mean?). If published, this will include your full peer review and any attached files.

If you choose “no”, your identity will remain anonymous but your review may still be made public.

**Do you want your identity to be public for this peer review?** For information about this choice, including consent withdrawal, please see our Privacy Policy.

Reviewer #2: No

[NOTE: If reviewer comments were submitted as an attachment file, they will be attached to this email and accessible via the submission site. Please log into your account, locate the manuscript record, and check for the action link "View Attachments". If this link does not appear, there are no attachment files.]

While revising your submission, please upload your figure files to the Preflight Analysis and Conversion Engine (PACE) digital diagnostic tool, https://pacev2.apexcovantage.com/. PACE helps ensure that figures meet PLOS requirements. To use PACE, you must first register as a user. Registration is free. Then, login and navigate to the UPLOAD tab, where you will find detailed instructions on how to use the tool. If you encounter any issues or have any questions when using PACE, please email PLOS at figures@plos.org. Please note that Supporting Information files do not need this step.

# Author comment: Author response to Decision Letter 1

**DOI:** 10.1371/journal.pone.0250010.r004

5 Mar 2021

Review Comments to the Author.

Please use the space provided to explain your answers to the questions above. You may also include additional comments for the author, including concerns about dual publication, research ethics, or publication ethics. (Please upload your review as an attachment if it exceeds 20,000 characters)

Reviewer #2. There are three main issues that must be clarified by the authors: Without clearly describing these issues it is impossible to know the representativeness of the sample and consequently, the value of the results obtained.

Response: Dear Reviewer, we recognize the limitations in our data, but nevertheless think that they are robust for the analyzed outcomes. Additionally, given the paucity of data on PCV impact from West Africa, with some of the highest pneumococcal disease burdens globally, we think our results are an important contribution to the literature.

Major comments

Comment #1. The samples studied and their distribution by year must be clear. It is impossible to know how many CSF samples of those obtained were studied each year, by what technique and their results. How many samples were from Yaounde or from outside each year of the study. This must be clearly depicted in Table 2.

Response # 1: We thank the Reviewer for this observation. Distinguishing the data between all samples and those from Yaoundéhas been provided in Table 2 under the column of "Number of CSF samples (no. of CSF within Yaoundé)”. [See table at end of this Major Comments section.]

In 2011, for example, 18 of the 47 CSF samples were collected from children living within Yaoundé. PCR was used as the final and confirmatory method for pathogen identification, the total number of confirmed S. pneumoniae is the same as that of PCR. Consequently, in this same year, pathogens were identified from 3 of 47 CSF samples, which were all S. pneumoniae: 1 pathogen was isolated via culture with PCR confirmationand 2 were identified by PCR only. 

Comment #2. Microbiological methods must be clearly described: what media were used for culture; what identification techniques were used, what antigen, what real-time PCR…

Response # 2: We do agree again with the Reviewer on the need to clearly demonstrate the aspect of reproducibility in scientific research in our methods. The following text hasbeen added under section 2.5.

Microbiological analysis of CSF specimens:

With regards to culture, 10 microliters of CSF were inoculated onto gentamicin blood agar and chocolate agar plates, and plates were incubated overnight at 370C in 5% carbon dioxide atmospheric conditions for 18 to 24 hours, as previously reported (Boula et al., 2019; Kwambana-Adams et al., 2016). After overnight culture, plates were examined for the respective characteristic morphological growth of pathogens, including S. pneumoniae, H. influenzae and N. meningitidis. Otherwise, for CSF specimens from hospitalized children with suspected bacterial infection, a rapid antigen test was used, BinaxNOWTM for pneumococcal identification or Pastorex for the other pathogens.

Colonies suspected to represent S. pneumoniae were confirmed by optochin susceptibility test (5µg optochin disc: Oxoid)with respect to the manufacturer's instructions. All confirmed isolates were placed in 1ml STGG tubes containing transport mediabroth with 16% glycerol. After treatment in the STGG tubes, the confirmed isolates were stored at temperatures of -700C, as were any CSF specimens where a confirmed isolate had not been obtained.Confirmed isolates and CSF specimens were shipped in dry ice (-700C) for further testing and serotyping at the World Health Organization Reference Regional Laboratory (WHO/RRL), Medical Research Council (MRC), The Gambia.

Serotyping of pneumococcal isolates:

At the WHO/RRL, real-time polymerase chain reaction (PCR) detection technique was used for serotyping. As earlier reported (Kwambana-Adams et al., 2016), RNAse P gene assay was applied on all CSF specimens to confirm that the samples were of human origin and to monitor the 

efficiency of amplification,which consisted of a series of cycles. First, there was an initial denaturation step at 950C for 10 minutes, followed by 45 cycles of 950C for 15 seconds and 600C for 60seconds. For each cycle of the targets, positivity was deduced using a cycle threshold (Ct) values, and Ct values of ≤36 were considered positive.

A CSF aliquot of 200µlwasadded to 50µl of TE buffer containing 0.08g/ml of lysozyme (Sigma, L-6876) and 150 U/ml of mutanolysin (Sigma, M-9901), and the mixture was incubated for 1 hour at 370C. Next, purified DNA extracts were exposed to asequential triplex quantitative PCR (qPCR) assay designed to detect 21 pneumococcal capsular serotypes/serogroups using the African scheme as earlier reported (Pimenta et al., 2013). Pneumococci with Ct values ≤32 by qPCR that could not be serotyped/serogrouped under this African scheme were serotyped byconventional multiplex serotyping PCR assays. (Serotype data for other pathogens were not available.)

Comment #3. How is it possible that “since 2012, uptake for the third dose of DTwP/PCV13 in Cameroon has been over 80% [16]” and in the study only 9.3% of the total number enrolled had a documented PCV13 vaccination history (5.5% with 3 doses)?

Response # 3: We again agree with the Reviewer that this might be a point of concern. We had taken accountof these limitations in the Discussion section.

Challenges with missing data (e.g., undocumented vaccination history) are a phenomenon in most of resource-limited countries, and electronic vaccination records are scarce.We were unable to capture vaccination data because most children(90.7%) had an undocumented vaccination status due to parents reporting to the hospital without vaccination cards/bookletswhen their children were sick.

The over 80% DTwP/PCV13 vaccination coverage reported in the text are administrative figures[16]. It is likely that most children have been immunized, even with an undocumented vaccination history, through bi-annual mass vaccination campaigns or supplementary immunization activities. In contrast to the DTwP and PCV13 vaccination coverage administrative figures, however, PCV13 vaccination history in this study was available only on 9.3% of subjects due to the retrospective nature of the effort to obtain that information. Children were identified with documented evidence of PCV13 vaccination through contact tracing, either by specific follow-up of vaccination status for cases that had been identified through routine IPD surveillance or by review of patients' medical records.

 

Table 2. Pathogens identified by culture (all pathogens) or other methods (pneumococcus only) from cerebrospinal fluid specimens of children aged 2─59 months from Cameroon. 

Year Streptococcus pneumoniae Haemophilus influenzae Neisseria meningitis Others 

Sub-set of CSF specimen analyzed Number of CSF specimens(no. of CSF within Yaoundé)

 Culture Antigen detection PCR* Culture Culture Culture 

2011 1 0 3 0 0 0 47 47 (18)

2012 5 0 5 3 2 4 62 276 (276)

2013 13 3 26 2 1 3 116 501 (473)

2014 8 2 16 3 1 4 154 662 (626)

2015 7 2 19 1 0 4 145 621 (563)

2016 6 1 15 4 0 5 149 633 (537)

2017 9 0 12 0 0 0 57 248 (207)

2018 6 0 7 0 1 1 35 143 (121)

Total 54 8 103 13 5 21 765 3131 (2821)

CSF = Cerebrospinal fluid; PCR = polymerase chain reaction; Others = (included E. coli, Shigella, Salmonella and Klebsiella species)*Because PCR was used as the final and confirmatory method for pathogen identification, the total number of confirmed S. pneumoniae is the same as that of PCR 

Fig 1. Profile of meningitis(purulent [Cases] and non-purulent [Non-cases]) in 2- to 59-month-old children hospitalized in Yaoundé, 2011 - 2018. 

Note: PCV13= 13-valent pneumococcal conjugate vaccine; CSF = cerebrospinal fluid; WBC = white blood cell; Cases = Purulent meningitis patients with a CSF WBC count ≥20 per mm3; Non-cases = Meningitis patients with a CSF WBC count <20 per mm3.

 

Other comments:

Comment #1. “We retrospectively analyzed laboratory-based surveillance data from August 2011 to December 2018.” However, Fig 1. represents data from 2011 to 2019. What happened in 2019? There were no cases of pneumococcal meningitis? It would be desirable to have the incidence data of pneumococcal meningitis of both periods studied, to see if the introduction of vaccine has represented a decrease in the incidence of meningitis or not.

Response # 1:We had partial data, but no serotyping of the CSF specimens, for the first quarter of 2019, so this was not reported. It was an error to have uploaded partial 2019 values in the Figure 1; however, this has been rectified and now reads Fig 1. represents data from 2011 to 2018.

We agree with the Reviewer on the need to have incidence data. We initially aimedto estimate incidence;however, the surveillance system was not population based and the completeness of event ascertainment could have varied from year to year.The lack of incidence data truly limited the extend of the analyses that we had planned originally.Likewise,many studies, even in developed countries, report data on proportions rather than incidence because of the difficulty in obtaining consistent data from a well-definedpopulation catchment area.

To address the Reviewer's concern, in the Discussion, we emphasized the need for improved surveillance to be able to document further the utility of PCV13 immunization in Cameroon. The over 80% DTwP/PCV13 vaccination coverage reported in the text are administrative figures [16]. It is likely that most children have been immunized, even with an undocumented vaccination history, through bi-annual mass vaccination campaigns or supplementary immunization activities. In contrast to the DTwP and PCV13 vaccination coverage administrative figures, however, PCV13 vaccination history in this study was available only on 9.3% of subjects due to the retrospective nature of the effort to obtain that information. Children were identified with documented evidence of PCV13 vaccination through contact tracing, either by specific follow-up of vaccination status for cases that had been identified through routine IPD surveillance or by review of patients' medical records.Another additional limitation of the PCV13 programme implemented in Cameroon is that it relies on an accelerated primary vaccination schedule with no booster.

Comment #2. The study population included children aged from 2 to 59 months during the period (August 2011 to December 2018) …Please, remove “(August 2011 to December 2018)” as it is already said 12 lines after.

Response # 2: We thank the Reviewer for drawing our attention here as well. This has been removed from the text.

Comment #3. If this is a retrospectively laboratory-based surveillance study, how were signed informed consents obtained from the parents/caretakers?

Response # 3:We thank the Reviewer for the chance to respond to this question. Yes, it was a retrospective, laboratory-based, surveillance study, and details are provided in the Methods section 2.1, which reads:Additionally, during the review of patients’ medical records and specific follow up of cases identified via routine IPD surveillance, vaccine history and signed informed consent forms were obtained from the parents/caretakers of each participating child.

As we also explained in the response to Major Comment #3, to retrieve comprehensive data (including vaccination history), we attempted to reach those parents/caretakers whose contact details were available in the registers. We reported what was found, being transparent in reporting exactly what proportion of children were identified with documented evidence of PCV13 vaccination through contact tracing, either by specific follow-up of vaccination status for cases that had been identified through routine IPD surveillance or by review of patients' medical records.

Comment # 4. The authors stated that “3-dose schedule (6, 10 and 14 weeks of age; 3+0) on date without any catch-up doses for older children” and later that “However, only children in the 2- to 11-month-old age bracket are eligible to receive free vaccination in the country.”

These two sentences are somehow contradictory; for instance, is a child of 6 months of age (24 weeks old) vaccinated or not?

Response # 4: We agree with the Reviewer that these two sentences might appear to be contradictory. Our intention here was to explain that, in the national immunization programme (NIP) in Cameroon, the age-group based approach differed between PCV13 and the traditional pediatric NIP vaccines. For the traditional pediatric NIP vaccines, children in the 2-to 11-month-old age group were eligible to receive free vaccination; whereas, for the PCV13 implementation programme,only those who were up to months age were eligible. Those older than 4 months were not vaccinated with PCV13. We observed this situation on the field were some children had their vaccination cards/booklets stamped with DTP1, DTP2 and DTP3 but were missing some doses of PCV13, although these were supposed to be administered simultaneously.

For clarification, we have complemented this statement with an additional sentence in Section 2.2, line 20 – 21 which now reads: ...although no catch-up vaccinations for PCV13 were planned for infants beyond 4months old.)

Comment # 5. Please, spell out Mother and Child Hospital (MCH) the first time it appears in the text.

Response # 5: This has been updated.

Comment # 6. Identification of S. pneumoniae from CSF by culture, …. Culture is not an identification technique. How were S. pneumoniae identified after cultured? Optochin test? Bili-solubility test?

Response # 6:We thank the Reviewer for drawing our attention to this, which has been revised in the text in section 2.5, lines 10-11with the following sentence:Colonies suspected to represent S. pneumoniae were confirmed using the optochin susceptibility test (5µg optochin disc; Oxoid) with respect to the manufacturer's instructions.

Comment # 7. Serotyping. The authors must include the serotyping real-time PCR used or at least a reference for the real-time PCR used for serotyping. How are serogroup 6 isolates identified? 6A/6B? 6A? What happens with serogroups 6C and 6D?

Response # 7: The text on serotyping has been revised (see section 2.6)

Serotyping of pneumococcal isolates

At the WHO/RRL, real-time polymerase chain reaction (PCR) detection technique was used for serotyping. As earlier reported (Kwambana-Adams et al., 2016), RNAse P gene assay was applied on all CSF specimens to confirm that the samples were of human origin and to monitor the 

efficiency of amplification, which consisted of a series of cycles. First, there was an initial denaturation step at 950C for 10 minutes, followed by 45 cycles of 950C for 15 seconds and 600C for 60 seconds. For each cycle of the targets, positivity was deduced using a cycle threshold (Ct) values, and Ct values of ≤36 were considered positive.

A CSF aliquot of 200 µl was added to 50 µl of TE buffer containing 0.08 g/ml of lysozyme (Sigma, L-6876) and 150 U/ml of mutanolysin (Sigma, M-9901), and the mixture was incubated for 1 hour at 370C. Next, purified DNA extracts were exposed to a sequential triplex quantitative PCR (qPCR) assay designed to detect 21 pneumococcal capsular serotypes/serogroups using the African scheme as earlier reported (Pimenta et al., 2013). Pneumococci with Ct values ≤32 by qPCR that could not be serotyped/serogrouped under this African scheme were serotyped by conventional multiplex serotyping PCR assays. (Serotype data for other pathogens were not available.)

Comment # 8. “Purulent meningitis was defined as suspected meningitis with a CSF white blood

cell (WBC) count ≥20 per mm3.” However, in reference 18 of the WHO they make the following definition: Leukocytosis (> 100 cells/mm3); Leukocytosis (10-100 cells/ mm3) AND either an elevated protein (>100 mg/dl) or decreased glucose (< 40 mg/dl).

Response # 8: We agree with the Reviewer with the widely used WHO case-definition which we had referenced. However, as is common throughout West Africa, CSF glucose and protein measurements were not available. 

To address the Reviewer's concern, we added the following text (see section 2.3 Case Definitions, lines 3 to 6):Purulent meningitis was defined as suspected meningitis with a CSF white blood cell (WBC) count ≥20/mm3". Although WHO also includes elevated protein or decreased glucose as part of the case definition, these tests were not routinely performed in Cameroon, as is true in much of West Africa.

Comment # 9. What antigen (trademark) was used for pneumococcal detection? Could all antigen positive CSF be typed by real-time PCR?

Response # 9: The antigen used for pneumococcal detection was BinaxNOWTM. This has been updated in the text, see section 2.5, line 9. 

We also wish to mention that the antigen tests used at the MCH or RRL (BinaxNOWTM or PASTOREXTM) do allow for all antigen-positive CSF to be typed by real-time PCR.

Comment # 10. During the early years of SURVAC only culture or antigen positive specimens were sent…Please, define “early years” (2011 to 2014?)

Response # 10: We thank the Reviewer for drawing our attention to this. We have updated this, and the sentence now reads: “During the first years (2011 to 2012) of SURVAC only culture or antigen positive specimens were sent…” (see section 2.5, line 17.)

Comment#11.Analyses were conducted with SPSS version 25.0.Please, include the developing corporation of this software.

Response # 11: This has been updated and now reads "Analyses were conducted using the International Business Machines Corporation (IBM) Statistical Package for Social Sciences (IBM SPSSStatistics 25.0)".(See section 2.7)

Comment # 12. “(Fig 1. Profile of Purulent meningitis (WBC counts in CSF) in 2-59 months old children hospitalized in Yaoundé: 2011 - 2018. Note: [PCV13= 13-valent pneumococcal conjugate vaccine; CSF = Cerebrospinal fluid; WBC = White blood cell])”. I suggest deleting all the text after Fig. 1 and place it in the space corresponding to figure legends and include the “Note” as a footnote of the figure. What is the meaning of IBD (secondary vertical axe?) Please, write the figure of cases among hospitalized patients (secondary axe) with two figures the symbol %. Add to the legend what does the blue line mean.

Response # 12: We thank the Reviewer for the suggestions provided with regards to Fig 1. We had formatted Fig. 1 initially as the Reviewer now suggests, but the editorial staff recommended that we follow Journal recommendations. We would be ready to modify this Figure again if the editorial staff deem it necessary.

As requested, "the blue line" (which is now red colored in the updated figure) represents the percentage of purulent meningitis cases among all hospitalized patients. For clarity, we have revised the secondary vertical axis to align with the case definition, purulent meningitis. Therefore, the red line represents the percentage of cases (purulent meningitis) among all hospitalized patients. All other modifications have been made as suggested by the Reviewer. [See table at end of this Major Comments section.]

Comment #13. Tables 1a and 1b. There are a lot of cases with missing clinical information, at least more than 50%: 1629 diagnosis unknown, 1617 undocumented outcome at discharged, 2840 vaccine status unknown…How representative is the study herein presented?

Response # 13: We again agree with the Reviewer on this. Our analyses were basically descriptive because, as noted in our response to Major Comment #3, challenges with missing data are a phenomenon in most of resource-limited countries. To address the Reviewer's concern, we have added in the Discussion as a limitation that: “missing data may have impacted representativeness of our findings. However, since most children in the study, and in Cameroon, are living under the poverty line, their risk factors are likely broadly similar for disease outcomes and vaccination status".

Comment #14. To which dates correspond the sub-set of CSF specimens (N=765) subsequently tested using either microbiological culture, antigen detection and/or polymerase chain reaction (PCR)? EPP or LPP? It is impossible to know the representativeness of the data if just 765/3131 cases (less than 25%) were studied unless they were homogenously distributed through the years of the study. How many cases was form Yaounde and how many from outside (Table 4)? How many were studied each year (or at least each period) from within/outside Yaounde? It is very strange that there were 103 cases of S. pneumoniae meningitis and only 5 cases of N. meningitidis meningitis.

Response # 14: The CSF specimens sub-set corresponds to the study period from August 2011 to December 2018. Because of limited logistic supplies the whole sets of specimens could not be analyzed; therefore, the method used was to randomly select a quarter of samples from each year for analyses. 

We have updated Table 2 with this information and explained elsewhere in the text that a representative subset of samples was chosen annually, for the years between 2012 and 2018. (By contrast, in the partial year2011, when PCV13 was introduced in July, all the available 47 CSF specimens were shipped to WHO/RRL.)

Comment # 15. “During the early years of SURVAC only culture or antigen positive specimens were sent to the WHO/RRL, but later all suspected isolates were shipped (Table 2).”

However in Table 2 in 2011 there are 3 PCR positive pneumococcal cases and only 1 by culture, and in 2013 there were 26 positives by PCR and only 13 by culture and 3 by antigen detection.

Response # 15: We agree with the Reviewer on the points been raised here. Specimens were sent as isolates (culture-positive) or CSF sample (antigen-positive). 

The practice at the WHO/RRL is that all isolates/samples received are tested. Firststep was to confirm if the specimens originate from humans. Secondly, all culture-positive isolates initially from Cameroon were to be confirmed with PCR. Since all isolates and samples were retested at the WHO/RRL, we have reported the PCR confirmed outcome as the final diagnostic method for pneumococcal detection. (Additional text has been added in the manuscript for clarity).

Comment # 16. The distribution of cases in Table 2 seems somehow heterogeneous (range 47-662 cases). Have the authors any explanation for this situation? Please comment in the results or in the discussion.

Response # 16: PCV13 programme entered Cameroon at the start of July 2011. Our study period spans from August 2011 to December 2018. (2011 was a partial year becausePCV13was introduced in July.) 

In the manuscript(Panel 2.), we have highlighted the lack of robustness in the surveillance programme in Cameroon to explain this heterogeneity. In particular, the surveillance programme performance was affected with factors/indicators such as:

1. Supply of reagents: Since the SURVAC project was not self-supported but relied entirely on stock supplies from partners, supplies were not usually timely and, in some cases, this delayed analyses.

2. Reporting and completion of entries: Because electronic data is not maintained, needing to track caregivers to get clinical/vaccine history was problematic. Hence, this contributed to the missing data scenario.

3. Consistency: The use of the international classification of diseases (ICD) codes is not mandatory in Cameroon, and this made it challenging to apply standard case definitions on clinical diagnosis.

Comment # 17. “The WHO/RRL in The Gambia did not use Quellung but rather PCR for serotyping, which in some cases did not distinguish individual serotypes (for example, 18A/18B/18C/18F).” However, in Table 3 serotype 18C is distinguished from other serogroup 18 serotypes. Also, serotype 6A/6B appear together but serotype 6A also appears alone.

Response # 17: We thank the Reviewer for this observation. As was earlier mentioned in Major Comment #2, this description that could include both serotypes and serogroups was a result of the method used. To recapitulate, purified DNA extracts were exposed to sequential triplex quantitative PCR (qPCR) assay designed to detect 21 pneumococcal capsular serotypes/serogroups using the African scheme as reported earlier(Pimenta et al., 2013).Pneumococci with Ct values ≤32 by qPCR that could not be serotyped/serogrouped under this African scheme were sequentially serotyped by conventional multiplex serotyping PCR assays. As the Reviewer notes, the WHO/RRL follows this Africa scheme approach.

Reaction no. Africa

1 1, 5, 23F

2 4, 6A/6B/6C/6D, 9V/9A

3 14, 18C/18A/18B/18F, 19F

4 3, 7F/7A, 19A

5 6C/6D, 12F/12A/12B/44/46, 22F/22A

6 15A/15F, 23A, 33F/33A/37

7 2, 11A/11D, 16F

As noted in the Major Comment #2 Response, “pneumococci with Ct values ≤32 by qPCR that could not be serotyped/serogrouped under this African scheme were serotyped by conventional multiplex PCR assays”.Therefore, 18C and 6A appear alone.

As mentioned for Major Comment #3, with respect to the vaccine uptake, likewise for the WHO / RRL output, we “reported what was found, being transparent in reporting exactly” the serotyping results that were provided by the laboratory (We have added as a footnote in Table 3 that:The serotypes that appear alone were done by PCR since the African scheme did not consistently yield a serotype.)

Comment # 18. “Of the 103 children with pneumococcal meningitis evaluated for serotype, a vaccine serotype was present in 62% (31/50) of cases in the EPE versus 35.8% (19/53) in the LPE (Table 3).” Please, include the statistic p-value for this comparison.

Response # 18: We have included the statistic p-value for this comparison and the sentence now reads:As shown in Table3, of the 103 children with pneumococcal meningitis evaluated for serotype, a vaccine serotype was present in 62% (31/50) of cases in the EPE versus 35.8% (19/53) in the LPE, showing a statistically significant difference between the two periods (p = 0.0081).

The statistical analysis method applied is described in Section 2.6 of the manuscript.

Comment # 19. “Table 4. Comparison of some baseline/serotype characteristics between the 'early and late post-PCV13 impact…” I suggest re-titling the Table as “Table 4. Comparison of some epidemiological characteristics and serotype distribution between the 'early and late post-PCV13 impact…” And replace “PCV13 Serotype status” with “PCV13 serotypes”.

Response # 19: We thank the Reviewer for the suggestions. Table 4 now reads, "Comparison of some epidemiological characteristics between the 'early and late post-PCV13 impact' periods of 2-to 59-month-old children with laboratory-confirmed pneumococcal meningitis hospitalized at the surveillance hospitals in Yaoundé (N=103): 2011 -2018".

Comment # 20. “Serotype-specific distribution differed between the EPE and LPE, respectively; 6A/6B (24%), 5 (6%) and 6A (6%) versus 14 (13.2%), 3 (7.6%), 4 (5.6%) and 18C (5.6%) (Table 4).” I would say that this distribution is depicted in Table 3 (but do not forget to include a reference to Table 4). Besides, are these differences statistically significant? Again, it is very strange to see the distribution of serogroup 6 isolates: 6A/6B and 6A alone… Does this mean that 6A/6B isolates are in fact serotype 6B isolates?

Response # 20: We thank the Reviewer again for drawing our attention to this. We have added in Table 3 two columns providing percentage difference estimates (with 95% confidence intervals) and the corresponding p-values. Table 4 has been referenced in the text as well.

With respect to the serogroup/serotype presentations, we have reported the serotype data as obtained from the WHO/RRL. While we agree with the Reviewer on the particularity of the distribution of isolates for serogroup 6 (and other serogroups), it reflects the application of the Africa scheme method. In the case of serogroup 6, for instance, Reaction no. 2 detects 6A/6B/6C/6D, while Reaction no. 4 detects 6C/6D. 

As mentioned for Major Comment #3, with respect to the vaccine uptake, likewise for the WHO/RRL output, we "reported what was found, being transparent in reporting exactly" the serotyping results that were provided by the laboratory. (We have added as a footnote in Table 3 that:"The serotypes that appear alone were done by PCR since the African scheme did not consistently yield a serotype".

Comment # 21. “Despite these gains, we did not see changes in the proportion of CSF samples with purulence in our study.” The presence of purulence can be due to multiple pathogens, not only to S. pneumoniae.

Response # 21: We agree with the Reviewer that purulence can be due to any of several different pathogens, including S. pneumoniae. We have revised the sentence and it now reads:“With respect to purulent meningitis, we did not see changes in the proportion of CSF samples with purulence in this study, although purulence can be due to multiple pathogens, not only S. pneumoniae.”

Comment # 22. “This may reflect trends in serotype-specific pneumococcal circulation related to confounding factors such as time since vaccination, varying population immunity, changes in antibiotic use patterns, or phenotypic shifts in organism invasiveness.” This sentence is quite speculative if not supported by an appropriate reference.

Response # 22: We agree again with the Reviewer on this and the statement has now been referenced (Oligbu et al., 2019; Rodrigo C et al., 2014)

Comment # 23. “However, as outlined in Table 2, because PCR was used for pathogen identification and serotyping, we used the total number of confirmed S. pneumoniae result as obtained by the PCR technique that has generally been used for molecular characterization [11,12,22].” The number of CSF sent and studied to the WHO/RRL each year must be clearly described, for instance in Table 2. It is not very understandable why purulent CSF negative for pneumococcal infection were not studied by PCR to detect N. meningitidis or H. influenzae and other pathogens in the WHO/RRL center.

Response # 23:We agree with the Reviewer on this as well. Clarity has been provided in Table 2 on the number of CSF sent to WHO/RRL each year. 

Funding remained a substantial challenge, which explains as well why only PCR was used. The WHO/RRL and SURVAC was substantially supported with funds from the United States Centers for Disease Control and Prevention, and when this was streamlined in 2011/2012, (coinciding with the time of IPD addition into the SURVAC network in August 2011) it obviously affected their capacity too. (We have added some text in the Discussion section, Panel 2).

Panel 2. Some of the observed indicators/factors influencing surveillance programme performance in Cameroon

1. Supply of reagents: Since the SURVAC project was not self-supported but relied entirely on stock supplies from partners, supplies were not usually timely and, in some cases, this delayed analyses.

2. Reporting and completion of entries: Because electronic data is not maintained, needing to track caregivers to get clinical/vaccine history was problematic. Hence, this contributed to the missing data scenario.

3. Consistency: The use of the international classification of diseases (ICD) codes is not mandatory in Cameroon, and this made it challenging to apply standard case definitions on clinical diagnosis.

Comment # 24.” Furthermore, in our study pathogen identification was not done routinely in a systematic fashion, which limited our ability to account appropriately for the sensitivity of each diagnostic method.”If pathogen identification was not done routinely, how can authors be sure that the decrease in VST or the increase in some VST is not due to a bias in the number of pathogens identified in each time-period?

Response # 24: Another good question from the Reviewer.

Pneumococcal carriage is a precursor to invasive pneumococcal disease (including meningitis), and the changes seen in carriage are most likely to be seen in disease. We had conducted a previous study on carriage on infants in this population and obtained similar trends. (Njuma Libwea J, Gröndahl-Yli-Hannuksela K, Kobela M, Toropainen M, Nyholm O, Ndombo PK, et al. Prevalence of pneumococcal nasopharyngeal colonization and serotypes circulating in Cameroonian children after the 13-valent pneumococcal conjugate vaccine introduction. Int J Infect Dis. 2020;98: 113–120. doi: 10.1016/j.ijid.2020.06.048).Although this cannot completely rule out the possibility of bias in the number of pathogens identified in each time-period, a decrease in the proportion of VST pneumococcal meningitis accompanied with a simultaneous increase in the proportion of NVST neumococcal meningitis is consistent with thisdocumented change in prevalence of pneumococcal serotype carriage in the era of conjugate vaccines.

Comment # 25. “By contrast, in Cameroon during the early years of the surveillance programme, only sentinel surveillance was performed.” What does this sentence mean? What kind of surveillance was done in the late years?

Response # 25:We thank the Reviewer for this observation. Our intention here was to state that while some other countries that initially started with sentinel surveillance have evolved to nation-wide surveillance, the situation has not changed in Cameroon since its inception (i.e., the early years). This sentence has now been revised and reads:By contrast, only sentinel surveillance has been performed in Cameroon since its

Attachment Submitted filename: Response to Reviewers_PONE-D-20-24718R1_PLoS_Libwea_05MAR21.docx Click here for additional data file.

# Peer review: Decision Letter 2

**DOI:** 10.1371/journal.pone.0250010.r005

**Authors:** Silvia Ricci

19 Mar 2021

PONE-D-20-24718R2

Impact of 13-valent pneumococcal conjugate vaccine on laboratory-confirmed pneumococcal meningitis and purulent meningitis among children ˂ 5 years in Cameroon,  2011 - 2018

PLOS ONE

Dear Dr. John Njuma Libwea,

Thank you for submitting your manuscript to PLOS ONE. After careful consideration, we feel that it has merit but does not fully meet PLOS ONE’s publication criteria as it currently stands. Therefore, we invite you to submit a revised version of the manuscript that addresses the points raised during the review process.

Please submit your revised manuscript by May 03 2021 11:59PM. If you will need more time than this to complete your revisions, please reply to this message or contact the journal office at plosone@plos.org. When you're ready to submit your revision, log on to https://www.editorialmanager.com/pone/ and select the 'Submissions Needing Revision' folder to locate your manuscript file.

Please include the following items when submitting your revised manuscript:

- A rebuttal letter that responds to each point raised by the academic editor and reviewer(s). You should upload this letter as a separate file labeled 'Response to Reviewers'.
- A marked-up copy of your manuscript that highlights changes made to the original version. You should upload this as a separate file labeled 'Revised Manuscript with Track Changes'.
- An unmarked version of your revised paper without tracked changes. You should upload this as a separate file labeled 'Manuscript'.

If you would like to make changes to your financial disclosure, please include your updated statement in your cover letter. Guidelines for resubmitting your figure files are available below the reviewer comments at the end of this letter.

If applicable, we recommend that you deposit your laboratory protocols in protocols.io to enhance the reproducibility of your results. Protocols.io assigns your protocol its own identifier (DOI) so that it can be cited independently in the future. For instructions see: http://journals.plos.org/plosone/s/submission-guidelines#loc-laboratory-protocols

We look forward to receiving your revised manuscript.

Kind regards,

Silvia Ricci

Academic Editor

PLOS ONE

Journal Requirements:

Please review your reference list to ensure that it is complete and correct. If you have cited papers that have been retracted, please include the rationale for doing so in the manuscript text, or remove these references and replace them with relevant current references. Any changes to the reference list should be mentioned in the rebuttal letter that accompanies your revised manuscript. If you need to cite a retracted article, indicate the article’s retracted status in the References list and also include a citation and full reference for the retraction notice.

[Note: HTML markup is below. Please do not edit.]

Reviewers' comments:

Reviewer's Responses to Questions

**Comments to the Author**

1. If the authors have adequately addressed your comments raised in a previous round of review and you feel that this manuscript is now acceptable for publication, you may indicate that here to bypass the “Comments to the Author” section, enter your conflict of interest statement in the “Confidential to Editor” section, and submit your "Accept" recommendation.

Reviewer #2: All comments have been addressed

**********

2. Is the manuscript technically sound, and do the data support the conclusions?

The manuscript must describe a technically sound piece of scientific research with data that supports the conclusions. Experiments must have been conducted rigorously, with appropriate controls, replication, and sample sizes. The conclusions must be drawn appropriately based on the data presented. 

Reviewer #2: Yes

**********

3. Has the statistical analysis been performed appropriately and rigorously? 

Reviewer #2: Yes

**********

4. Have the authors made all data underlying the findings in their manuscript fully available?

The PLOS Data policy requires authors to make all data underlying the findings described in their manuscript fully available without restriction, with rare exception (please refer to the Data Availability Statement in the manuscript PDF file). The data should be provided as part of the manuscript or its supporting information, or deposited to a public repository. For example, in addition to summary statistics, the data points behind means, medians and variance measures should be available. If there are restrictions on publicly sharing data—e.g. participant privacy or use of data from a third party—those must be specified.

Reviewer #2: Yes

**********

5. Is the manuscript presented in an intelligible fashion and written in standard English?

PLOS ONE does not copyedit accepted manuscripts, so the language in submitted articles must be clear, correct, and unambiguous. Any typographical or grammatical errors should be corrected at revision, so please note any specific errors here.

Reviewer #2: Yes

**********

6. Review Comments to the Author

Please use the space provided to explain your answers to the questions above. You may also include additional comments for the author, including concerns about dual publication, research ethics, or publication ethics. (Please upload your review as an attachment if it exceeds 20,000 characters)

Reviewer #2: All questions raised by this reviewer have been properly answered and I actually appreciate the effort made by the authors in improving the text with the new comments introduced.

I only have 3 minor comments:

1. Abstract. “…EPE were 6A/6B (24%), 5 (6%) and 6A (6%),…”

As not all serogroup 6 could be typed to the serotype level, I suggest (only for the abstract) to add 6A + 6A/B serotypes, what should make a 6A/6B (30%) and delete 6A (6%).

As both serotypes 6A/6B are included in the PCV13 and many molecular tests don’t differentiate between them, the reading of the abstract will be easier. In the text, with the explanations given, the results can be left as they are, differentiating between 6A and 6A/6B serotypes.

2. Page 6. “Pneumococci with Ct values ≤32 by qPCR that could not be serotyped/serogrouped under this African scheme were serotyped by conventional multiplex serotyping PCR assays.”

Please, add a reference for the conventional multiplex serotyping PCR assays used.

3. Page 15. “We had considered this as a potential source of bias to our findings, but as documented with the prevalence of pneumococcal carriage in the era of conjugate vaccines, our data is consistent with the trends of decrease in some VST accompanied with a simultaneous increase in some NVST.”

Please, provide a reference for the prevalence of pneumococcal carriage in the era of conjugate vaccines.

**********

7. PLOS authors have the option to publish the peer review history of their article (what does this mean?). If published, this will include your full peer review and any attached files.

If you choose “no”, your identity will remain anonymous but your review may still be made public.

**Do you want your identity to be public for this peer review?** For information about this choice, including consent withdrawal, please see our Privacy Policy.

Reviewer #2: No

[NOTE: If reviewer comments were submitted as an attachment file, they will be attached to this email and accessible via the submission site. Please log into your account, locate the manuscript record, and check for the action link "View Attachments". If this link does not appear, there are no attachment files.]

While revising your submission, please upload your figure files to the Preflight Analysis and Conversion Engine (PACE) digital diagnostic tool, https://pacev2.apexcovantage.com/. PACE helps ensure that figures meet PLOS requirements. To use PACE, you must first register as a user. Registration is free. Then, login and navigate to the UPLOAD tab, where you will find detailed instructions on how to use the tool. If you encounter any issues or have any questions when using PACE, please email PLOS at figures@plos.org. Please note that Supporting Information files do not need this step.

# Author comment: Author response to Decision Letter 2

**DOI:** 10.1371/journal.pone.0250010.r006

21 Mar 2021

PONE-D-20-24718R1

Impact of 13-valent pneumococcal conjugate vaccine on laboratory-confirmed pneumococcal meningitis and purulent meningitis among children ˂ 5 years in Cameroon, 2011 - 2018

PLOS ONE

Dear Dr.John Njuma Libwea, 

Thank you for submitting your manuscript to PLOS ONE. After careful consideration, we feel that it has merit but does not fully meet PLOS ONE’s publication criteria as it currently stands. Therefore, we invite you to submit a revised version of the manuscript that addresses the points raised during the review process.

Please submit your revised manuscript by May 03 2021 11:59PM. If you will need more time than this to complete your revisions, please reply to this message or contact the journal office at plosone@plos.org. When you're ready to submit your revision, log on to https://www.editorialmanager.com/pone/ and select the 'Submissions Needing Revision' folder to locate your manuscript file.

Please include the following items when submitting your revised manuscript:

• A rebuttal letter that responds to each point raised by the academic editor and reviewer(s). You should upload this letter as a separate file labeled 'Response to Reviewers'.

• A marked-up copy of your manuscript that highlights changes made to the original version. You should upload this as a separate file labeled 'Revised Manuscript with Track Changes'.

• An unmarked version of your revised paper without tracked changes. You should upload this as a separate file labeled 'Manuscript'.

If you would like to make changes to your financial disclosure, please include your updated statement in your cover letter. Guidelines for resubmitting your figure files are available below the reviewer comments at the end of this letter.

If applicable, we recommend that you deposit your laboratory protocols in protocols.io to enhance the reproducibility of your results. Protocols.io assigns your protocol its own identifier (DOI) so that it can be cited independently in the future. For instructions see: http://journals.plos.org/plosone/s/submission-guidelines#loc-laboratory-protocols

We look forward to receiving your revised manuscript.

Kind regards,

Silvia Ricci

Academic Editor

PLOS ONE________________________________________

Review Comments to the Author.

Please use the space provided to explain your answers to the questions above. You may also include additional comments for the author, including concerns about dual publication, research ethics, or publication ethics. (Please upload your review as an attachment if it exceeds 20,000 characters)

Reviewer #2: All questions raised by this reviewer have been properly answered and I actually appreciate the effort made by the authors in improving the text with the new comments introduced.

I only have 3 minor comments:

________________________________________

Response: Dear Reviewer, we thank you for the appreciations and recognize the minor comments you have raised in our data. Additionally, given the paucity of data on PCV impact from West Africa, with some of the highest pneumococcal disease burdens globally, we think our results are an important contribution to the literature.

Minor comments

Comment #1. Abstract. “…EPE were 6A/6B (24%), 5 (6%) and 6A (6%),…”

As not all serogroup 6 could be typed to the serotype level, I suggest (only for the abstract) to add 6A + 6A/B serotypes, what should make a 6A/6B (30%) and delete 6A (6%).

As both serotypes 6A/6B are included in the PCV13 and many molecular tests don’t differentiate between them, the reading of the abstract will be easier. In the text, with the explanations given, the results can be left as they are, differentiating between 6A and 6A/6B serotypes.

Response # 1: We thank the Reviewer for this suggestion, and this has now been revised in the Abstract to read; " The most frequent pneumococcal meningitis VSTs during the EPE were 6A/6B (30%) and 5 (6%), and during the LPE were 14 (13.2%), 3 (7.6%), 4 (5.6%) and 18C (5.6%)".

Comment #2. Page 6. “Pneumococci with Ct values ≤32 by qPCR that could not be serotyped/serogrouped under this African scheme were serotyped by conventional multiplex serotyping PCR assays.” Please, add a reference for the conventional multiplex serotyping PCR assays used.

Response # 2: The corresponding reference to this has now been added (See Section 2.6, line 14).

Comment # 3. Page 15. “We had considered this as a potential source of bias to our findings, but as documented with the prevalence of pneumococcal carriage in the era of conjugate vaccines, our data is consistent with the trends of decrease in some VST accompanied with a simultaneous increase in some NVST.” Please, provide a reference for the prevalence of pneumococcal carriage in the era of conjugate vaccines.

Response # 3: The references text have been added (See Discussion section, paragraph, line 51).

Attachment Submitted filename: Response to Reviewers_PONE-D-20-24718R1_20 MAR21.docx Click here for additional data file.

# Peer review: Decision Letter 3

**DOI:** 10.1371/journal.pone.0250010.r007

**Authors:** Silvia Ricci

30 Mar 2021

Impact of 13-valent pneumococcal conjugate vaccine on laboratory-confirmed pneumococcal meningitis and purulent meningitis among children ˂ 5 years in Cameroon,  2011 - 2018

PONE-D-20-24718R3

Dear Dr.John Njuma Libwea,

We’re pleased to inform you that your manuscript has been judged scientifically suitable for publication and will be formally accepted for publication once it meets all outstanding technical requirements.

Within one week, you’ll receive an e-mail detailing the required amendments. When these have been addressed, you’ll receive a formal acceptance letter and your manuscript will be scheduled for publication.

An invoice for payment will follow shortly after the formal acceptance. To ensure an efficient process, please log into Editorial Manager at http://www.editorialmanager.com/pone/, click the 'Update My Information' link at the top of the page, and double check that your user information is up-to-date. If you have any billing related questions, please contact our Author Billing department directly at authorbilling@plos.org.

If your institution or institutions have a press office, please notify them about your upcoming paper to help maximize its impact. If they’ll be preparing press materials, please inform our press team as soon as possible -- no later than 48 hours after receiving the formal acceptance. Your manuscript will remain under strict press embargo until 2 pm Eastern Time on the date of publication. For more information, please contact onepress@plos.org.

Kind regards,

Silvia Ricci

Academic Editor

PLOS ONE

Additional Editor Comments (optional):

Reviewers' comments:

Reviewer's Responses to Questions

**Comments to the Author**

1. If the authors have adequately addressed your comments raised in a previous round of review and you feel that this manuscript is now acceptable for publication, you may indicate that here to bypass the “Comments to the Author” section, enter your conflict of interest statement in the “Confidential to Editor” section, and submit your "Accept" recommendation.

Reviewer #2: All comments have been addressed

**********

2. Is the manuscript technically sound, and do the data support the conclusions?

The manuscript must describe a technically sound piece of scientific research with data that supports the conclusions. Experiments must have been conducted rigorously, with appropriate controls, replication, and sample sizes. The conclusions must be drawn appropriately based on the data presented. 

Reviewer #2: Yes

**********

3. Has the statistical analysis been performed appropriately and rigorously? 

Reviewer #2: Yes

**********

4. Have the authors made all data underlying the findings in their manuscript fully available?

The PLOS Data policy requires authors to make all data underlying the findings described in their manuscript fully available without restriction, with rare exception (please refer to the Data Availability Statement in the manuscript PDF file). The data should be provided as part of the manuscript or its supporting information, or deposited to a public repository. For example, in addition to summary statistics, the data points behind means, medians and variance measures should be available. If there are restrictions on publicly sharing data—e.g. participant privacy or use of data from a third party—those must be specified.

Reviewer #2: Yes

**********

5. Is the manuscript presented in an intelligible fashion and written in standard English?

PLOS ONE does not copyedit accepted manuscripts, so the language in submitted articles must be clear, correct, and unambiguous. Any typographical or grammatical errors should be corrected at revision, so please note any specific errors here.

Reviewer #2: Yes

**********

6. Review Comments to the Author

Please use the space provided to explain your answers to the questions above. You may also include additional comments for the author, including concerns about dual publication, research ethics, or publication ethics. (Please upload your review as an attachment if it exceeds 20,000 characters)

Reviewer #2: (No Response)

**********

7. PLOS authors have the option to publish the peer review history of their article (what does this mean?). If published, this will include your full peer review and any attached files.

If you choose “no”, your identity will remain anonymous but your review may still be made public.

**Do you want your identity to be public for this peer review?** For information about this choice, including consent withdrawal, please see our Privacy Policy.

Reviewer #2: No

# Editor report: Acceptance letter

**DOI:** 10.1371/journal.pone.0250010.r008

**Authors:** Silvia Ricci

5 Apr 2021

PONE-D-20-24718R3 

Impact of 13-valent pneumococcal conjugate vaccine on laboratory-confirmed pneumococcal meningitis and purulent meningitis among children ˂5 years in Cameroon, 2011 - 2018 

Dear Dr. Njuma Libwea:

I'm pleased to inform you that your manuscript has been deemed suitable for publication in PLOS ONE. Congratulations! Your manuscript is now with our production department. 

If your institution or institutions have a press office, please let them know about your upcoming paper now to help maximize its impact. If they'll be preparing press materials, please inform our press team within the next 48 hours. Your manuscript will remain under strict press embargo until 2 pm Eastern Time on the date of publication. For more information please contact onepress@plos.org.

If we can help with anything else, please email us at plosone@plos.org. 

Thank you for submitting your work to PLOS ONE and supporting open access. 

Kind regards, 

PLOS ONE Editorial Office Staff

on behalf of

Dr. Silvia Ricci 

Academic Editor

PLOS ONE
